# Intelligent Method of Identifying the Nonlinear Dynamic Model for Helicopter Turboshaft Engines

**DOI:** 10.3390/s24196488

**Published:** 2024-10-09

**Authors:** Serhii Vladov, Arkadiusz Banasik, Anatoliy Sachenko, Wojciech M. Kempa, Valerii Sokurenko, Oleksandr Muzychuk, Piotr Pikiewicz, Agnieszka Molga, Victoria Vysotska

**Affiliations:** 1 Department of Scientific Work Organization and Gender Issues, Kremenchuk Flight College of Kharkiv National University of Internal Affairs, 17/6, Peremohy Street, 39605 Kremenchuk, Ukraine; serhii.vladov@univd.edu.ua; 2 Department of Mathematical Methods in Technics and Informatics, Silesian University of Technology, 44-100 Gliwice, Poland; wojciech.kempa@polsl.pl (W.M.K.); piotr.pikiewicz@polsl.pl (P.P.); 3 Research Institute for Intelligent Computer Systems, West Ukrainian National University, 11 Lvivska Street, 46009 Ternopil, Ukraine; as@wunu.edu.ua; 4 Department of Teleinformatics, Kazimierz Pulaski University of Radom, 26-600 Radom, Poland; a.molga@uthrad.pl; 5 Department of Information Systems and Technologies, Kharkiv National University of Internal Affairs, 27, L. Landau Avenue, 61080 Kharkiv, Ukraine; rector_hnuvs@ukr.net (V.S.); o.muzychuk23@gmail.com (O.M.); 6 Information Systems and Networks Department, Lviv Polytechnic National University, 12, Bandera Street, 79013 Lviv, Ukraine; victoria.a.vysotska@lpnu.ua; 7 Institute of Computer Science, Osnabrück University, 1, Friedrich-Janssen-Street, 49076 Osnabrück, Germany

**Keywords:** helicopter turboshaft engines, dynamic model, identifying, engine starting and acceleration, Elman recurrent neural network with dynamic stack memory, training, accuracy, sensors

## Abstract

This research focused on the helicopter turboshaft engine dynamic model, identifying task solving in unsteady and transient modes (engine starting and acceleration) based on sensor data. It is known that about 85% of helicopter turboshaft engines operate in steady-state modes, while only around 15% operate in unsteady and transient modes. Therefore, developing dynamic multi-mode models that account for engine behavior during these modes is a critical scientific and practical task. The dynamic model for starting and acceleration modes has been further developed using on-board parameters recorded by sensors (gas-generator rotor r.p.m., free turbine rotor speed, gas temperature in front of the compressor turbine, fuel consumption) to achieve a 99.88% accuracy in identifying the dynamics of these parameters. An improved Elman recurrent neural network with dynamic stack memory was introduced, enhancing the robustness and increasing the performance by 2.7 times compared to traditional Elman networks. A theorem was proposed and proven, demonstrating that the total execution time for *N* Push and Pop operations in the dynamic stack memory does not exceed a certain value *O*(*N*). The training algorithm for the Elman network was improved using time delay considerations and Butterworth filter preprocessing, reducing the loss function from 2.5 to 0.12% over 120 epochs. The gradient diagram showed a decrease over time, indicating the model’s approach to the minimum loss function, with optimal settings ensuring the stable training.

## 1. Introduction

### 1.1. Relevance of the Research

Modern aviation technologies require high-precision and reliable control systems to ensure the stable operation of helicopters in various operating conditions [1]. The key to such system components is the helicopter turboshaft engine (TE), on which not only flight efficiency, but also safety depends [2]. To expand the control process range, an urgent scientific and practical task is to develop helicopter TE dynamic multi-mode models that take into account the peculiarities of engine behavior and its operation in unsteady transient modes. The helicopter TE dynamic model’s identification is a complex task that requires taking into account many factors and high modeling accuracy [3]. This circumstance requires that the existing identification methods either improve so that they can be applied to helicopter TE non-stationary dynamic models or the development of completely new methods.

The identifying gas-turbine engine parameters (including helicopter TE) in existing methods are mainly focused on linear stationary models and have many limitations and disadvantages that make them difficult to use for non-stationary models [4,5]. This creates a need to develop new approaches for helicopter TEs representing nonlinear models and methods for their identification.

### 1.2. State-of-the-Art

To determine the gas-turbine engines’ mathematical model parameters, a traditional linking method is used [6]. In this method, parameter determination is carried out by employing two thermogas-dynamic tasks: the direct and inverse one. The direct task evaluates the gas-turbine engines’ (including the helicopter TE) mathematical model adequacy to a real object before and after identification, and the inverse task determines the characteristics of the gas-turbine engines’ components.

In addition to the traditional linking method, formal identification methods are widely used [7,8]. These enable one to directly find the parameter values or relative corrections to the original parameters, depending on the stated aim function. When identifying the gas-turbine engines’ (including helicopter TE) mathematical model, formal methods solve two tasks: a direct one and an optimization task [9]. The direct task determines the parameters (residuals, influence coefficients, etc.) of the calculated operating modes, which are then used in the optimization task to find the parameter values or corrections to the initial parameters in order to minimize the objective function.

Identification methods such as the adjustment method, least moduli and quadratic weighted approximations method, method using the Huber function, nonlinear programming and optimization methods, and other similar approaches involve varying efficiency parameters included in the engine units’ mathematical models and are not suitable for solving the identification task [10].

Identification methods, such as frequency methods, identification by the moment’s method, identification with random input signals, consider typical effects on the identification object to obtain experimental data and analyze them [11]. Frequency methods use harmonic influence at the motor input to obtain amplitude-phase characteristics, which limits their application [12].

The fuzzy set method [13] is used to analyze mathematical models with heterogeneous a priori information, the particular criteria and restrictions specified in a fuzzy manner requiring consideration [14]. In [15], the least squares method for parameter identification and alternative techniques was discussed. Disadvantages of this method include the significant deviations in estimates for small deviations in the initial data, a weak robustness, and limitations in taking into account a priori information [16]. These shortcomings have led to the search for more advanced estimation methods such as improving the least squares computational algorithm, regularizing the problem for correctness, or using measures that increase the quality criterion robustness [17].

One of the directions for improving the least squares method is based on the system matrix singular value decomposition (SVD) method [18], which is used to calculate pseudoinverse matrices.

The experience of other scientists includes the use of genetic programming [19,20], neural network models [21,22,23], neuro-fuzzy models [24,25,26], frequency methods [27], Kalman filter [28,29,30], wavelet transforms [31], and optimization methods [32,33]. These approaches offer diverse strategies for handling nonlinearity and uncertainty in gas turbine engine dynamics, providing flexibility and adaptability for various applications. By leveraging advanced computational techniques, researchers [19,20,21,22,23,24,25,26,27,28,29,30,31,32,33] have been able to enhance the models’ precision and robustness, ultimately contributing to more efficient and reliable engine performance in operational environments.

In [34], neural network models for identifying the gas-turbine engines dynamic model based on changes in fuel consumption (pressure, temperature, frequency) were developed. In [35], a recurrent neural network constructing method for the gas-turbine engines’ model identifying was proposed. Its key disadvantage is ensuring reliable and stable operation in the FADEC closed loop control in dynamic modes. In [35], a data-driven fault diagnosis system for gas-turbine engine faults was developed using hybrid multi-mode machine learning strategies, improving diagnostic accuracy and reliability.

Taking into account the modern requirements for the quality of transient processes, the existing identification methods do not always provide the required quality and are designed for steady-state operating conditions. Unsteady mode modeling is important for further synthesis and the development of an automatic control system (ACS) for gas-turbine engines (including helicopter TEs) [36,37,38]. Neural network-based methods use modern computers with a performance of more than 10 billion operations per second to represent the GTE model as a “black box” with a neural network model and a large number of tunable coefficients [39,40,41]. The identification task is to construct a model that describes the changes in the engine parameters when the input influence and external conditions change [42,43].

Nowadays about 85% of gas-turbine engines (including helicopter TEs) operate in steady-state modes, and only about 15% in unsteady and transient modes (in the starting and acceleration modes). To expand control, it is necessary to develop dynamic multi-mode models that take into account the engine behavior in unsteady and transient modes. The existing identification methods have limitations, and efficiency improvement of the dynamic model identifying process of gas-turbine engines (including helicopter TEs) based on helicopter flight operation data is required [44,45].

The choice of neural networks to develop the helicopter TE dynamic model is driven by their ability to capture complex nonlinear relations and temporal dependencies in engine dynamics [46], which are difficult for traditional methods like fuzzy logic and genetic algorithms. Recurrent neural networks [47,48] are particularly suited for handling time-series data, enabling accurate predictions of engine behavior across a wide range of transitional modes [49]. While fuzzy logic handles uncertainty, it struggles with the high dimensionality of helicopter TE dynamics [50], and genetic algorithms, though effective for optimization, are less efficient for real-time modeling [51]. The ability of neural network’ to train from data, self-adjust through backpropagation, and generalize across conditions makes them ideal for real-time on-board engine monitoring systems [52]. They provide a more robust, scalable solution, ensuring continuous adaptation to varying conditions, essential for reliable helicopter TE operation in dynamic flight environments [53]. This adaptability further reinforces their suitability for real-time applications where safety and performance are paramount. 

Based on the review of the above-mentioned related works in the helicopter TE dynamic model identification field, it was established that the research carried out in [34], which relied on the traditional Elman network, was the closest analogy. In [34], the Elman neural network, with its structure and training algorithm based on the gradient steepest descent method, allowed them to solve the dynamic identification issue, achieving accuracy that was 1.5 times higher compared to the least square’s method, and 2 times higher than in noisy conditions. However, the key drawback of the Elman network—its limited capacity for the long-term memory and handling complex temporal dependencies—remains. This requires network improvement through the introduction of dynamic stack memory to ensure the reliable storage of temporal data as well as enhance stability in the engine changing dynamic operating modes.

Thus, to solve this issue, we propose a method that takes into account the control object dynamic properties of a nonlinear nature. The feasibility of using a neural network approach was justified by the fact that it does not require a priori knowledge about the system. Moreover, it copes effectively with multi-mode and parallel failure scenarios, which is significantly superior to traditional methods. 

## 2. Materials and Methods

### 2.1. Development of the Helicopter Turboshaft Engine Nonlinear Dynamic Model

#### 2.1.1. Development of the Helicopter Turboshaft Engine Nonlinear Dynamic Model at Engine Starting Mode

Currently, there are no adequate helicopter TE nonlinear dynamic models for starting mode that are suitable for creating closed control loops [54,55]. Therefore, before developing a method for such model parameter identification, it is necessary to determine its mathematical form. Due to significant changes in the engine characteristics from the moment the combustion chamber is ignited until the idle state is reached [56], the dynamic model must be nonlinear. It is assumed that this model is represented by a differential equation system in Cauchy normal form for rotor speeds, and algebraic equations for other engine parameters. In this form, the dynamic model representation form can be used for the synthesis and adjustment of the helicopter TE ACS, and will serve as the basis for obtaining the propulsion parameters’ transfer functions through analytical methods. Unlike the acceleration mode, at the starting mode, there is no line of steady-state modes, which eliminates the model constructing possibility in “deviations” from the “operating line”. Thus, the above considerations require presenting a helicopter TE nonlinear dynamic model at the starting mode in the following mathematical form:

(1) $$f_{1}(t)=A_{1}(t)\;\cdot \;n_{TC}(t)+B_{1}(t)\;\cdot \;n_{FT}(t)+C_{1}(t)\;\cdot \;T_{G}^{*}(t)+D_{1}(t)\;\cdot \;u(t),\;\ldots \;f_{N}(t)=A_{N}(t)\;\cdot \;n_{TC}(t)+B_{N}(t)\;\cdot \;n_{FT}(t)+C_{N}(t)\;\cdot \;T_{G}^{*}(t)+D_{N}(t)\;\cdot \;u(t),$$

where *N* is the model equation number; *f*_1_(*t*), *f*_2_(*t*) are nonlinear vector functions; *n_TC_*(*t*) is the gas-generator rotor r.p.m. value recorded on-board the helicopter (D-2M sensor is used); *n_FT_*(*t*) is the free turbine rotor speed value, recorded on-board the helicopter (a D-1M sensor was used); $T_{G}^{*}$(*t*) is the gas temperature in front of the compressor turbine value, recorded on-board the helicopter (a sensor consisting of 14 T-101 thermocouples was used) [56,57]; *u*(*t*) is the control action vector; *A*_1_(*t*)…*A_N_*(*t*), *B*_1_(*t*)…*B_N_*(*t*), *C*_1_(*t*)…*C_N_*(*t*), *D*_1_(*t*)…*D_N_*(*t*) are the differential and algebraic equations’ coefficient matrices.

To take into account the influence of the external conditions (flight altitude *h* = *h*(*t*), flight speed *v* = *v*(*t*), *M* = *M*(*t*) is the Mach number at flight altitude *h* = *h*(*t*), *ρ* = *ρ*(*t*) is the air density at flight altitude *h* = *h*(*t*)), system (1) is represented in the given coordinates.

The nonlinear nature of Equation (1) manifests in the engine equations’ parameters and coefficients dependence on time. In a more general case, this can be any parameter that determines the engine state at each starting moment (a certain parameter α is taken). The model (1) general form must be clarified in each specific case [57]. Then, the helicopter TE model at starting mode will look like this:

(2) $$\frac{\partial n_{TC}}{\partial \alpha }=a_{11}\left(\alpha \right)\cdot n_{TC}\left(\alpha \right)+a_{12}\left(\alpha \right)\cdot n_{FT}\left(\alpha \right)+a_{13}\left(\alpha \right)\cdot T_{G}^{*}\left(\alpha \right)+b_{1}\left(\alpha \right)\cdot G_{T}\left(\alpha \right),\;\frac{\partial n_{FT}}{\partial \alpha }=a_{21}\left(\alpha \right)\cdot n_{TC}\left(\alpha \right)+a_{22}\left(\alpha \right)\cdot n_{FT}\left(\alpha \right)+a_{23}\left(\alpha \right)\cdot T_{G}^{*}\left(\alpha \right)+b_{2}\left(\alpha \right)\cdot G_{T}\left(\alpha \right),\;\frac{\partial T_{G}^{*}}{\partial \alpha }=a_{31}\left(\alpha \right)\cdot n_{TC}\left(\alpha \right)+a_{32}\left(\alpha \right)\cdot n_{FT}\left(\alpha \right)+a_{33}\left(\alpha \right)\cdot T_{G}^{*}\left(\alpha \right)+b_{3}\left(\alpha \right)\cdot G_{T}\left(\alpha \right).$$

The theoretical basis for representing the engine model in starting mode in forms (1) and (2) is Kolmogorov’s theorem, which solves the fundamental mathematical problem of representing some functions through others. To identify the helicopter TE dynamic model presentation in starting mode, we proposed the use of a representation form in forms (1) and (2). Thus, the structure and form of the helicopter TE dynamic model presentation in starting mode were developed, where the model parameters were presented as functional dependencies on the mode parameter.

The initial information for developing a method for the helicopter TE nonlinear dynamic models’ parameters at starting mode determining, based on the proposed approach to solving the identification task, is the helicopter TE nonlinear mathematical model structure and experimental data recorded during normal engine operation. Due to significant changes in engine characteristics from the moment the combustion chamber is ignited to idle mode, the dynamic model must be nonlinear. It is assumed that the helicopter TE dynamic model at starting mode can be represented by a differential equations system in the normal Cauchy form for rotor speeds, and algebraic equations for other propulsion parameters [58].

The parameters *n_TC_*(*α*), *n_FT_*(*α*), $T_{G}^{*}$(*α*), and *G_T_*(*α*) are the helicopter TE corresponding parameter values obtained experimentally, depending on the mode parameter *α*. The mode parameter *α* is understood as a certain value that allows us to determine the “distance” from the combustion chamber ignition moment to the starting mode considered moment. In the simplest case, this may be the time that has passed since the fuel ignited.

The identifying task of the helicopter TE nonlinear dynamic model at starting mode is to determine the model (2) coefficients *a_ij_*(*α*) functional dependencies. To solve this, a method was proposed that is, following stages or procedures, a systematic set aimed at determining the model (2) coefficients *a_ij_*(*α*), which includes the preliminary processing of experimental data, their low-frequency filtering, the engine parameters obtaining functional dependencies on time, these parameter values and the first derivatives calculation with respect to the rotor speed, the algebraic equations overdetermined the systems’ formation and solution as well as the dynamic equation coefficients determining the formulation of tasks as quadratic programming tasks with a subsequent solution. As these stages result, the identified model’s coefficient arrays are formed, and their functional dependencies on the mode parameter are determined.

**Figure 1:**
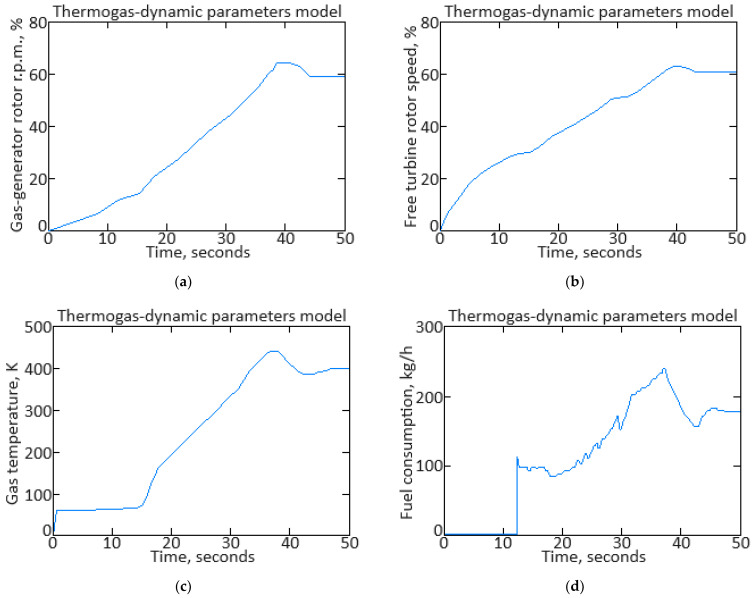
Diagrams of changes in the helicopter turboshaft engine parameters (using for example, the TV3-117 engine) at starting mode: (**a**) fuel consumption, (**b**) gas-generator rotor r.p.m., (**c**) free turbine rotor speed, (**d**) gas temperature in front of the compressor turbine (author’s research).

At the first stage of identification, the initial data are prepared in order to ensure their suitability for subsequent use. Registration systems used on engine test benches, as a rule, have a significant number of drawbacks; in most cases, the data contain interference, failures, repeated data, information omissions, and a significant noise level. The registration system’s source files are translated into text format and processed using appropriate software implemented in the computing environment of the MATLAB software package. Figure 1 presents the initial data for the identification procedure in the helicopter TE parameters in diagram form. The recording frequency of the helicopter TE parameters obtained was 15.75 Hz [59]. The engine was started according to the timing diagram [60].

**Figure 2:**
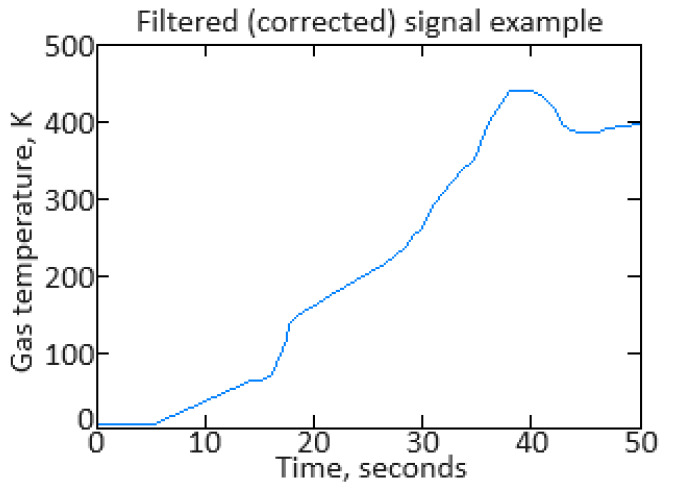
Diagram of changes in the helicopter turboshaft engine’s (using for example, the TV3-117 engine) gas temperature in front of the compressor turbine after applying the low-frequency filtering procedure at starting mode (author’s research).

In addition to eliminating the registration system’s shortcomings, the original information, as a rule, requires high-frequency interference filtering from information and measuring channels. At the second stage of the identification procedure, low-pass filters are created. Practice shows that the most suitable filters for this purpose are filters with an infinite impulse response, for example, Butterworth filters. However, such filters have a significant phase shift of the filtered signal. To eliminate this, filtering must be performed twice: first in the forward direction, and then in the reverse direction [61]. Figure 2 shows the low-pass filtering result with an eighth-order Butterworth filter for the gas temperature in front of the compressor turbine parameter.

At the third stage, the parameters’ functional dependencies, as presented in Figure 1, are determined from the time elapsed since the combustion chamber was started (mode parameter *α*). The required functional dependencies are necessary to determine the model “working line” and to calculate the first-order derivatives with respect to the rotation frequency of both rotors. The derivatives values are needed for the left-hand side numerical values for the model (2) equations. The specificity of the experimental data leads to the need for their approximation, either by a neural network or a cubic spline, which is a *n*-th complex order interpolating polynomials [62].

At the fourth stage, the helicopter TE parameter values are calculated based on their functional dependencies on mode parameter *α*. At this stage, calculations are also performed to calculate the first derivative values with respect to the gas-generator rotor r.p.m. and the free turbine rotor speed.

At the fifth stage, the linear vector-matrix equation coefficients forming model (2) are determined. For this purpose, an experimental data matrix **A** = [*n_TC_*, *n_FT_*, $T_{G}^{*}$, *G_T_*] with dimensions (*m* × 4), where the value *m* is the discrete sample number in the given experimental data, is formed. Next, the coefficient vectors are formed that need to be determined: *x*_1_ = [*a*_11_, *a*_12_, *a*_13_], *x*_2_ = [*a*_21_, *a*_22_, *a*_23_], *x*_3_ = [*a*_31_, *a*_32_, *a*_33_]. After this, vectors are compiled from the rotation frequencies’ derivative values and the helicopter TE’s other parameter values $B_{1}=\frac{\partial n_{TC}}{\partial \alpha }$, $B_{2}=\frac{\partial n_{FT}}{\partial \alpha }$, $B_{3}=\frac{\partial T_{G}^{*}}{\partial \alpha }$. Three equations for overdetermined systems are compiled and solved:

(3) $$\left\{\begin{array}{c} A\cdot x_{1}=B_{1}\\ A\cdot x_{2}=B_{2}\\ A\cdot x_{3}=B_{3} \end{array}\right.$$

The solution of system (3) is performed by using the standard method of minimizing the squared residuals sum. Many mathematical programs use an overridden left matrix division operator to do this. As a result of solving system (3), the element values of vectors *x*_1_, *x*_2_, *x*_3_ are determined.

The values of the dynamic model equations’ coefficient vectors, obtained at the fifth stage of the identification procedure, represent the model parameters’ rough “average” estimate, realizing its linear representation. To make the dynamic starting model nonlinear, it is necessary to change the model coefficients, depending on the current state of the launch process. One of the possible ways to solve this task is to determine the Equation (2) coefficients’ functional dependence on a parameter characterizing the engine’s current state during the starting process, for example, the time interval value from the moment the combustion chamber is ignited. For unification purposes, this parameter, which can take different values characterizing the current engine state, is called mode parameter *α*. Thus, the result at this stage is the representation of the helicopter TE model in the form:

(4) $$\frac{\partial n_{TC,i}}{\partial \alpha_{i}}=a_{11,i}\left(\alpha_{i}\right)\cdot n_{TC,i}\left(\alpha_{i}\right)+a_{12,i}\left(\alpha_{i}\right)\cdot n_{FT,i}\left(\alpha_{i}\right)+a_{13,i}\left(\alpha_{i}\right)\cdot T_{G,i}^{*}\left(\alpha_{i}\right)+b_{1,i}\left(\alpha_{i}\right)\cdot G_{T,i}\left(\alpha_{i}\right),\;\frac{\partial n_{FT,i}}{\partial \alpha_{i}}=a_{21,i}\left(\alpha_{i}\right)\cdot n_{TC,i}\left(\alpha_{i}\right)+a_{22,i}\left(\alpha_{i}\right)\cdot n_{FT,i}\left(\alpha_{i}\right)+a_{23,i}\left(\alpha_{i}\right)\cdot T_{G,i}^{*}\left(\alpha_{i}\right)+b_{2,i}\left(\alpha_{i}\right)\cdot G_{T,i}\left(\alpha_{i}\right),\;\frac{\partial T_{G,i}^{*}}{\partial \alpha_{i}}=a_{31,i}\left(\alpha_{i}\right)\cdot n_{TC,i}\left(\alpha_{i}\right)+a_{32,i}\left(\alpha_{i}\right)\cdot n_{FT,i}\left(\alpha_{i}\right)+a_{33,i}\left(\alpha_{i}\right)\cdot T_{G,i}^{*}\left(\alpha_{i}\right)+b_{3,i}\left(\alpha_{i}\right)\cdot G_{T,i}\left(\alpha_{i}\right),\;n_{TC,i+1}=n_{TC,i}\left(\alpha_{i}\right)+h\cdot \frac{\partial n_{TC,i}}{\partial \alpha_{i}},\;n_{FT,i+1}=n_{FT,i}\left(\alpha_{i}\right)+h\cdot \frac{\partial n_{FT,i}}{\partial \alpha_{i}},\;T_{G,i+1}^{*}=T_{G,i}^{*}\left(\alpha_{i}\right)+h\cdot \frac{\partial T_{G,i}^{*}}{\partial \alpha_{i}},$$

where *a_ij_*_,0_ are constants determined as the result of the linear Equation (3) solving systems, *h* is the time sampling step value in the registration files, and *i* is the integration step number.

Given the structure of the mathematical model, at each integration step, it is necessary to calculate the equations’ coefficient values so that the calculated engine output parameter is as close as possible to its experimental value. As a “closeness” measure of calculated and experimental processes, we proposed the use of an objective function in quadratic form. The use of the quadratic form can be justified by the following provisions: quadratic objective functions are unimodal, that is, they only have one extremum point; a quadratic programming task without restrictions has an analytical solution, which allows one to check the numerical solution; and, finally, quadratic programming theory is one of the most nonlinear programming developed branches, and its numerical methods are well-known and have been comprehensively tested [62].

Therefore, at the sixth stage, a search is made for the coefficient values of the equations that are optimal for each value of the parameter in starting mode. For the differential equations from the coefficient values from Equation (4), the determining task is formulated as a quadratic programming task with objective function *F*(*x*_1_) to determine the differential equation coefficients based on the gas-generator rotor r.p.m., *F*(*x*_2_) to determine the differential equation coefficients based on the free turbine rotor speed, and *F*(*x*_3_) to determine the differential equation coefficients for the gas temperature in front of the compressor turbine:

(5) $$F\left(x_{1}\right)={\left(n_{TC}\left(x_{1}\right)-n_{TC}^{ref}\left(\alpha_{i}\right)\right)}^{2},\;F\left(x_{2}\right)={\left(n_{FT}\left(x_{2}\right)-n_{FT}^{ref}\left(\alpha_{i}\right)\right)}^{2},\;F\left(x_{3}\right)={\left(T_{G}^{*}\left(x_{3}\right)-T_{G}^{*ref}\left(\alpha_{i}\right)\right)}^{2}.$$

As an objective function *F*(*x*_1_) minimizing result, the coefficients *a*_11_, *a*_12_, *a*_13_, *b*_1_ are determined for the dynamics parameter *α_i_*. Similarly, as an objective function *F*(*x*_2_) minimizing result, the coefficients *a*_21_, *a*_22_, *a*_23_, *b*_2_ are determined, and as an objective function *F*(*x*_3_) minimizing result, the coefficients *a*_31_, *a*_32_, *a*_33_, *b*_3_ are determined. The optimization task is solved for all *α* values from the initial value to the last with step *h*. The initial values for determining the required coefficients are the vector *x*_1_ = [*a*_11_, *a*_12_, *a*_13_]^T^, *x*_2_ = [*a*_21_, *a*_22_, *a*_23_]^T^, *x*_3_ = [*a*_31_, *a*_32_, *a*_33_]^T^ elements, obtained at the fifth stage.

Using the example of objective function *F*(*x*_1_), it can be shown that the objective functions under consideration are convex and unimodal, that is, their minimum is achieved only at one point. For this, the objective function *F*(*x*_1_) is written as:

(6) $$F\left(x_{1}\right)={\left(n_{TC}\left(x_{1}\right)-n_{TC}^{ref}\left(\alpha_{i}\right)\right)}^{2}=F\left(x_{1}\right)={\left(n_{TC}\left(a_{11},a_{12},a_{13},b_{1}\right)-n_{TC}^{ref}\left(\alpha_{i}\right)\right)}^{2}\;={\left(a_{11,i}\left(\alpha_{i}\right)\cdot n_{TC,i}\left(\alpha_{i}\right)+a_{12,i}\left(\alpha_{i}\right)\cdot n_{FT,i}\left(\alpha_{i}\right)+a_{13,i}\left(\alpha_{i}\right)\cdot T_{G,i}^{*}\left(\alpha_{i}\right)+b_{1,i}\left(\alpha_{i}\right)\cdot G_{T,i}\left(\alpha_{i}\right)-n_{TC}^{ref}\left(\alpha_{i}\right)\right)}^{2}\;=\left(a_{11,i}\left(\alpha_{i}\right)\cdot n_{TC,i}\left(\alpha_{i}\right)\right)\cdot (a_{11,i}\left(\alpha_{i}\right)\cdot n_{TC,i}\left(\alpha_{i}\right)+a_{12,i}\left(\alpha_{i}\right)\cdot n_{FT,i}\left(\alpha_{i}\right)+a_{13,i}\left(\alpha_{i}\right)\cdot T_{G,i}^{*}\left(\alpha_{i}\right)+b_{1,i}\left(\alpha_{i}\right)\cdot G_{T,i}\left(\alpha_{i}\right)\;-n_{TC}^{ref}\left(\alpha_{i}\right))+\left(a_{12,i}\left(\alpha_{i}\right)\cdot n_{FT,i}\left(\alpha_{i}\right)\right)\cdot (a_{11,i}\left(\alpha_{i}\right)\cdot n_{TC,i}\left(\alpha_{i}\right)+a_{12,i}\left(\alpha_{i}\right)\cdot n_{FT,i}\left(\alpha_{i}\right)+a_{13,i}\left(\alpha_{i}\right)\cdot T_{G,i}^{*}\left(\alpha_{i}\right)\;+b_{1,i}\left(\alpha_{i}\right)\cdot G_{T,i}\left(\alpha_{i}\right)-n_{TC}^{ref}\left(\alpha_{i}\right))+\left(a_{13,i}\left(\alpha_{i}\right)\cdot T_{G,i}^{*}\left(\alpha_{i}\right)\right)\cdot \left(a_{11,i}\left(\alpha_{i}\right)\cdot n_{TC,i}\left(\alpha_{i}\right)+a_{12,i}\left(\alpha_{i}\right)\cdot n_{FT,i}\left(\alpha_{i}\right)\right.\;\left.+a_{13,i}\left(\alpha_{i}\right)\cdot T_{G,i}^{*}\left(\alpha_{i}\right)+b_{1,i}\left(\alpha_{i}\right)\cdot G_{T,i}\left(\alpha_{i}\right)-n_{TC}^{ref}\left(\alpha_{i}\right)\right)+\left(b_{1,i}\left(\alpha_{i}\right)\cdot G_{T,i}\left(\alpha_{i}\right)\right)\cdot (a_{11,i}\left(\alpha_{i}\right)\cdot n_{TC,i}\left(\alpha_{i}\right)\;+a_{12,i}\left(\alpha_{i}\right)\cdot n_{FT,i}\left(\alpha_{i}\right)+a_{13,i}\left(\alpha_{i}\right)\cdot T_{G,i}^{*}\left(\alpha_{i}\right)+b_{1,i}\left(\alpha_{i}\right)\cdot G_{T,i}\left(\alpha_{i}\right)-n_{TC}^{ref}\left(\alpha_{i}\right)-n_{TC}^{ref}\left(\alpha_{i}\right)\cdot (a_{11,i}\left(\alpha_{i}\right)\cdot n_{TC,i}\left(\alpha_{i}\right)\;+a_{12,i}\left(\alpha_{i}\right)\cdot n_{FT,i}\left(\alpha_{i}\right)+a_{13,i}\left(\alpha_{i}\right)\cdot T_{G,i}^{*}\left(\alpha_{i}\right)+b_{1,i}\left(\alpha_{i}\right)\cdot G_{T,i}\left(\alpha_{i}\right)-n_{TC}^{ref}\left(\alpha_{i}\right)).$$

Quadratic form (6) is represented in vector-matrix form as:

(7) $$F={\left(\begin{array}{c} a_{11}\\ a_{12}\\ a_{13}\\ b_{1} \end{array}\right)}^{\prime }\cdot \left(\begin{array}{cccc} n_{TC}^{2} & n_{TC}\cdot n_{FT} & n_{TC}\cdot T_{G}^{*} & n_{TC}\cdot G_{T}\\ n_{TC}\cdot n_{FT} & n_{FT}^{2} & n_{FT}\cdot T_{G}^{*} & n_{FT}\cdot G_{T}\\ n_{TC}\cdot T_{G}^{*} & n_{FT}\cdot T_{G}^{*} & {\left(T_{G}^{*}\right)}^{2} & T_{G}^{*}\cdot G_{T}\\ n_{TC}\cdot G_{T} & n_{FT}\cdot G_{T} & {T_{G}^{*}\cdot G}_{T} & G_{T}^{2} \end{array}\right)\cdot \left(\begin{array}{c} a_{11}\\ a_{12}\\ a_{13}\\ b_{1} \end{array}\right)+{\left(\begin{array}{c} -2\cdot n_{TC}\cdot n_{TC}^{ref}\\ -2\cdot n_{FT}\cdot n_{TC}^{ref}\\ -2\cdot T_{G}^{*}\cdot n_{TC}^{ref}\\ -2\cdot G_{T}\cdot n_{TC}^{ref} \end{array}\right)}^{\prime }\cdot \left(\begin{array}{c} a_{11}\\ a_{12}\\ a_{13}\\ b_{1} \end{array}\right)+{\left(n_{TC}^{ref}\right)}^{2}.$$

Let us present (7) in standard form:

(8) $$F\left(x\right)=x^{\prime }\cdot C\cdot x+p^{\prime }\cdot x+{\left(n_{TC}^{ref}\right)}^{2},$$

where

(9) $$x=\left(\begin{array}{c} a_{11}\\ a_{12}\\ a_{13}\\ b_{1} \end{array}\right),\;C=\left(\begin{array}{cccc} n_{TC}^{2} & n_{TC}\cdot n_{FT} & n_{TC}\cdot T_{G}^{*} & n_{TC}\cdot G_{T}\\ n_{TC}\cdot n_{FT} & n_{FT}^{2} & n_{FT}\cdot T_{G}^{*} & n_{FT}\cdot G_{T}\\ n_{TC}\cdot T_{G}^{*} & n_{FT}\cdot T_{G}^{*} & {\left(T_{G}^{*}\right)}^{2} & T_{G}^{*}\cdot G_{T}\\ n_{TC}\cdot G_{T} & n_{FT}\cdot G_{T} & {T_{G}^{*}\cdot G}_{T} & G_{T}^{2} \end{array}\right),\;p=\left(\begin{array}{c} -2\cdot n_{TC}\cdot n_{TC}^{ref}\\ -2\cdot n_{FT}\cdot n_{TC}^{ref}\\ -2\cdot T_{G}^{*}\cdot n_{TC}^{ref}\\ -2\cdot G_{T}\cdot n_{TC}^{ref} \end{array}\right).$$

Matrix *C* is positive definite because the values on its main diagonal are always positive, which is positive definiteness [62] sign. In other words, matrix *C* vanishes only when all variables *a*_11_, *a*_12_, *a*_13_, *b*_1_ vanish, and is positive if at least one of these variables is non-zero.

A strictly defined matrix is non-singular, therefore it has an inverse matrix *C*^−1^. The aim function *F*(*x*_1_) unconditional minimum, denoted as *x_opt_*, is easily determined from the equation:

grad(*F*(*x*)) = *p* + 2 · *C* · *x* = 0, (10)

where

(11) $$x_{opt}=-\frac{1}{2}\cdot C^{-1}\cdot p.$$

Thus, the proposed local objective functions’ strict convexity and the presence of a global minimum are proven.

It is advisable to minimize the objective function *F*(*x*_1_) by using one of the direct unconditional optimization methods, for example, using the Nelder–Mead method (deformed polyhedron method or simplex method) [63,64,65], which allows for solutions to be found for problems with a large number of variables and is more universal than the calculation using relation (11).

Thus, as a result of the sequence solving of the optimization tasks, all of the equations’ coefficient numerical value arrays that make up the engine model can be determined.

The seventh stage is the formation of the functional dependencies of the helicopter TE dynamic model’s coefficient values on parameter *α*, which are determined based on the cubic spline interpolation or neural network approximation. These dependencies are necessary to calculate the starting processes using the identified model.

Thus, a method was developed for the identification task for the helicopter TE nonlinear dynamic model, in starting mode, based on experimental data, which uses the experimental data cubic spline approximation (neural network approximation) and represents the identification problem as a quadratic programming tasks sequence, where the objective functions are local optimality criteria, which together allows for high accuracy when determining the model parameters.

#### 2.1.2. Development of the Helicopter Turboshaft Engine Nonlinear Dynamic Model in Engine Acceleration Mode

The closed channel feature for controlling the throttle response mode is controlled based on the derivative program value of the helicopter TE gas-generator rotor r.p.m. The approach to modeling injectivity proposed by [62] is based on the set use of linear models that describe the engine dynamic characteristics in the small vicinity of static modes but suffers from gaps in the helicopter TE derived parameter values when connecting linear models. To overcome these tasks, there is a need for an engine mathematical model with new representation in the throttle response mode, which will allow us to synthesize and debug a closed control loop. Therefore, there is a need to identify helicopter TE nonlinear dynamic models in acceleration mode. As is known, the dynamic processes for a helicopter TE gas-generator rotor r.p.m. are described by a differential equation [62]:

(12) $$J\cdot \frac{\partial \omega }{\partial t}=M_{T}\left(n_{TC},G_{T}\right)-M_{k}\left(n_{TC}\right)=\delta M\left(n_{TC},G_{T}\right),$$

where *J* is the rotor inertia moment, $\omega =\frac{\pi \cdot n_{TC}}{30}$ is the engine rotor rotation angular speed, *M_T_*(*n_TC_*, *G_T_*) is the gas-generator driving moment, *M_k_*(*n_TC_*) is the gas-generator resistance moment, and *δM*(*n_TC_*, *G_T_*) is the excess torque.

For a twin-shaft helicopter TE (for example, TV3-117), Equation (12) is valid for each of the two rotors. It is only necessary to take into account that there is a gas-dynamic connection between the rotors, so a change in the rotation speed in one will cause a change in the rotation speed of the other [62]. In addition, the gas temperature in front of the compressor turbine affects the gas-dynamic connection between the helicopter TE twin-shaft rotors, since a change in temperature leads to a change in the pressure and gas flow speed, which, in turn, causes a change in the speed of each rotor rotation. Taking this into account, the equations of the helicopter TE dynamics will look like:

(13) $$\frac{\partial n_{TC}}{\partial t}=\frac{30}{\pi \cdot J_{1}}\cdot \delta M_{1}\left(n_{TC},{n_{FT},T_{G}^{*},G}_{T}\right)=f_{1}\left(n_{TC},{n_{FT},T_{G}^{*},G}_{T}\right),\;\frac{\partial n_{FT}}{\partial t}=\frac{30}{\pi \cdot J_{2}}\cdot \delta M_{2}\left(n_{TC},{n_{FT},T_{G}^{*},G}_{T}\right)=f_{2}\left(n_{TC},{n_{FT},T_{G}^{*},G}_{T}\right),$$

where $\frac{\partial n_{TC}}{\partial t}$ is the gas-generator rotor r.p.m. acceleration and $\frac{\partial n_{FT}}{\partial t}$ is the free turbine rotor speed acceleration.

The dynamic model requirement being developed is as follows: along with dynamic processes, the model must reproduce stationary engine operating modes in the range from low throttle to emergency [62]. To take this requirement into account, we introduced the functional dependences of the helicopter TE parameters on mode parameter *α*, which will uniquely determine the point on the static mode line from idle mode to emergency mode: *n_TC_*(α), *n_FT_*(α), $T_{G}^{*}$(α) и *G_T_*(α). These dependencies ensure the accurate modeling of both transient and steady-state behaviors across the entire operating spectrum.

Then, expressions (13) according to [62] will take the form:

(14) $$\frac{\partial n_{TC}}{\partial t}=f_{1}\left(n_{TC}\left(\alpha \right),{n_{FT}\left(\alpha \right),T_{G}^{*}\left(\alpha \right),G}_{T}\left(\alpha \right)\right),\;\frac{\partial n_{FT}}{\partial t}=f_{2}\left(n_{TC}\left(\alpha \right),{n_{FT}\left(\alpha \right),T_{G}^{*}\left(\alpha \right),G}_{T}\left(\alpha \right)\right).$$

By expanding the right-hand sides of expression (14) into a Taylor series, we obtained [18,66]:

(15) $$\frac{\partial n_{TC}}{\partial t}=\frac{\partial n_{TC}\left(\alpha \right)}{\partial n_{TC}}+∆n_{TC}\cdot \frac{\partial n_{TC}\left(\alpha \right)}{\partial n_{TC}}+∆n_{FT}\cdot \frac{\partial n_{TC}\left(\alpha \right)}{\partial n_{FT}}+∆T_{G}^{*}\cdot \frac{\partial n_{TC}\left(\alpha \right)}{\partial T_{G}^{*}}+∆G_{T}\cdot \frac{\partial n_{TC}\left(\alpha \right)}{\partial G_{T}},\;\frac{\partial n_{FT}}{\partial t}=\frac{\partial n_{FT}\left(\alpha \right)}{\partial n_{TC}}+∆n_{TC}\cdot \frac{\partial n_{FT}\left(\alpha \right)}{\partial n_{TC}}+∆n_{FT}\cdot \frac{\partial n_{FT}\left(\alpha \right)}{\partial n_{FT}}+∆T_{G}^{*}\cdot \frac{\partial n_{FT}\left(\alpha \right)}{\partial T_{G}^{*}}+∆G_{T}\cdot \frac{\partial n_{FT}\left(\alpha \right)}{\partial G_{T}},\;\frac{\partial T_{G}^{*}}{\partial t}=\frac{\partial T_{G}^{*}\left(\alpha \right)}{\partial n_{TC}}+∆n_{TC}\cdot \frac{\partial T_{G}^{*}\left(\alpha \right)}{\partial n_{TC}}+∆n_{FT}\cdot \frac{\partial T_{G}^{*}\left(\alpha \right)}{\partial n_{FT}}+∆T_{G}^{*}\cdot \frac{\partial T_{G}^{*}\left(\alpha \right)}{\partial T_{G}^{*}}+∆G_{T}\cdot \frac{\partial T_{G}^{*}\left(\alpha \right)}{\partial G_{T}},\;∆n_{TC}=n_{TC}-n_{TC}\left(\alpha \right),\;∆n_{FT}=n_{FT}-n_{FT}\left(\alpha \right),\;∆T_{G}^{*}=T_{G}^{*}-T_{G}^{*}\left(\alpha \right),\;∆G_{T}=G_{T}-G_{T}\left(\alpha \right).$$

Based on Equation (15), a helicopter TE nonlinear model in deviations was obtained:

(16) $$∆\left(\frac{\partial n_{TC}}{\partial t}\right)=a_{11}\left(\alpha \right)\cdot ∆n_{TC}+a_{12}\left(\alpha \right)\cdot ∆n_{FT}+a_{13}\left(\alpha \right)\cdot ∆T_{G}^{*}+b_{1}\left(\alpha \right)\cdot ∆G_{T},\;∆\left(\frac{\partial n_{FT}}{\partial t}\right)=a_{21}\left(\alpha \right)\cdot ∆n_{TC}+a_{22}\left(\alpha \right)\cdot ∆n_{FT}+a_{23}\left(\alpha \right)\cdot ∆T_{G}^{*}+b_{2}\left(\alpha \right)\cdot ∆G_{T},\;∆\left(\frac{\partial T_{G}^{*}}{\partial t}\right)=a_{31}\left(\alpha \right)\cdot ∆n_{TC}+a_{32}\left(\alpha \right)\cdot ∆n_{FT}+a_{33}\left(\alpha \right)\cdot ∆T_{G}^{*}+b_{3}\left(\alpha \right)\cdot ∆G_{T},\;∆n_{TC}=n_{TC}-n_{TC}\left(\alpha \right),\;∆n_{FT}=n_{FT}-n_{FT}\left(\alpha \right),\;∆T_{G}^{*}=T_{G}^{*}-T_{G}^{*}\left(\alpha \right),\;∆G_{T}=G_{T}-G_{T}\left(\alpha \right).$$

**Figure 3:**
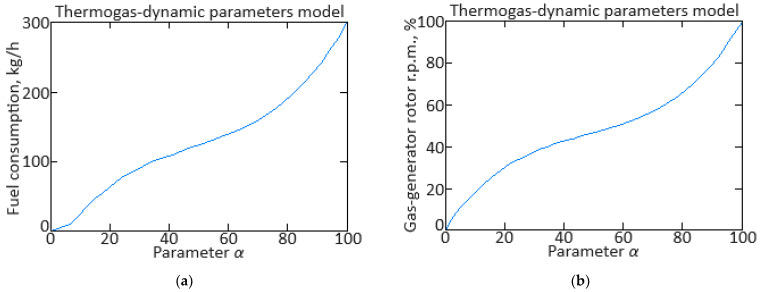
The dependency diagrams of the helicopter turboshaft engine parameters: (**a**) fuel consumption, (**b**) gas-generator rotor r.p.m. (author’s research).

**Figure 4:**
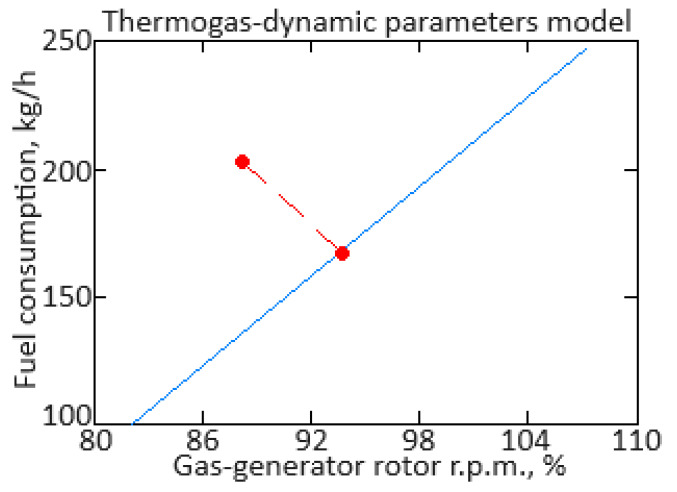
The statistical characteristics of the helicopter turboshaft engine parameters (author’s research).

From Equation (16), it follows that to identify the model parameters, it is necessary to determine the values of the coefficients *a_ij_*(*α*). To determine the *α* value at the transient process of each moment, the procedure presented in [62,67] was applied, according to which, based on the fuel consumption *G_T_*(*α*) and gas-generator rotor r.p.m. *n_TC_*(*α*) dependences on the mode parameter *α* in static modes (Figure 3), a static mode line was constructed on the dynamic characteristic (Figure 4).

In addition, in Figure 4, point 1, with coordinates (*n_TC_*, *G_T_*), reflects the current values of the helicopter TE parameters during the acceleration process, and point 2, with coordinates (*n_TC_*(*α*), *G_T_*(*α*)), reflects the static mode line point closest to point 1. As stated in [62], the proximity measure of points 1 and 2 is the “distance” between them:

(17) $$d\left(\alpha \right)=\sqrt{{\left(n_{TC}-n_{TC}\left(\alpha \right)\right)}^{2}+{\left(G_{T}-G_{T}\left(\alpha \right)\right)}^{2}}.$$

The mode parameter *α* value is determined from this minimum distance condition, which leads to the formation of an optimization task with an optimality criterion in the objective function form:

(18) $$F\left(\alpha \right)=\sqrt{{\left(n_{TC}-n_{TC}\left(\alpha \right)\right)}^{2}+{\left(G_{T}-G_{T}\left(\alpha \right)\right)}^{2}}\to min,$$

in this case, the objective function (18) is positive definite and has a global minimum point. The solution to the objective function (18) minimizing task is performed using one of the one-dimensional search methods, for example, the golden section method [62,68,69,70]. As a result, the mode parameter value is determined, which is necessary to obtain the model coefficients *a_ij_*(*α*) and the static dependencies *n_TC_*(*α*), *n_FT_*(*α*), $T_{G}^{*}$(*α*), and *G_T_*(*α*) values.

Thus, the structure and description of the helicopter TE nonlinear dynamic model form in static and unsteady modes in the nonlinear differential system form and algebraic equations have been proposed. A distinctive feature of the model is the presentation of its parameters in the functional dependencies on the mode parameter form, which ensures that the dynamic processes are nonlinear in nature and the derivatives show continuity with respect to the engine parameters. The numerical values of the model parameters are calculated from these functional dependencies, based on the optimization task solving. Applying the method described for the helicopter TE model in starting mode, we obtained:

(19) $$∆\left(\frac{\partial n_{TC,i}}{\partial \alpha_{i}}\right)=a_{11,i}\cdot ∆n_{TC}\left(\alpha_{i}\right)+a_{12,i}\cdot ∆n_{FT}\left(\alpha_{i}\right)+a_{13,i}\cdot ∆T_{G}^{*}\left(\alpha_{i}\right)+b_{1,i}\cdot ∆G_{T}\left(\alpha_{i}\right),\;∆\left(\frac{\partial n_{FT,i}}{\partial \alpha_{i}}\right)=a_{21,i}\cdot ∆n_{TC}\left(\alpha_{i}\right)+a_{22,i}\cdot ∆n_{FT}\left(\alpha_{i}\right)+a_{23,i}\cdot ∆T_{G}^{*}\left(\alpha_{i}\right)+b_{2,i}\cdot ∆G_{T}\left(\alpha_{i}\right),\;∆\left(\frac{\partial T_{G,i}^{*}}{\partial \alpha_{i}}\right)=a_{31,i}\cdot ∆n_{TC}\left(\alpha_{i}\right)+a_{32,i}\cdot ∆n_{FT}\left(\alpha_{i}\right)+a_{33,i}\cdot ∆T_{G}^{*}\left(\alpha_{i}\right)+b_{3,i}\cdot ∆G_{T}\left(\alpha_{i}\right),\;∆n_{TC,i+1}=n_{TC,i}\left(\alpha_{i}\right)+h\cdot ∆\left(\frac{\partial n_{TC,i}}{\partial \alpha_{i}}\right),\;∆n_{FT,i+1}\left(\alpha_{i}\right)=n_{FT,i}\left(\alpha_{i}\right)+h\cdot \left(\frac{\partial n_{FT,i}}{\partial \alpha_{i}}\right),\;∆T_{G,i+1}^{*}\left(\alpha_{i}\right)=T_{G,i}^{*}\left(\alpha_{i}\right)+h\cdot \left(\frac{\partial T_{G,i}^{*}}{\partial \alpha_{i}}\right).$$

Thus, a search was made for the coefficient values of the equations for the helicopter TE mathematical model given the structure at the acceleration mode, which were optimal at each integration step. To determine the equations from the coefficient values in Equation (19), their determination task was formulated as a quadratic programming task with objective function *F*(*x*_1_) to determine the differential equation coefficients of the gas-generator rotor r.p.m., *F*(*x*_2_) to determine the differential equation coefficients of the free turbine rotor speed, and *F*(*x*_3_) to determine the differential equation coefficients of the gas temperature in front of the compressor turbine:

(20) $$F\left(x_{1}\right)={\left(n_{TC}\left(x_{1}\right)-n_{TC}^{ref}\left(\alpha_{i}\right)\right)}^{2},\;F\left(x_{2}\right)={\left(n_{FT}\left(x_{2}\right)-n_{FT}^{ref}\left(\alpha_{i}\right)\right)}^{2},\;F\left(x_{3}\right)={\left(T_{G}^{*}\left(x_{3}\right)-T_{G}^{*ref}\left(\alpha_{i}\right)\right)}^{2},$$

where *x*_1_ = [*a*_11,*i*_, *a*_12,*i*_, *a*_13,*i*_], *x*_2_ = [*a*_21,*i*_, *a*_22,*i*_, *a*_23,*i*_], *x*_3_ = [*a*_31,*i*_, *a*_32,*i*_, *a*_33,*i*_].

The feature of the optimization tasks under consideration is the use of the initial values of the coefficients from well-known linear models. This is necessary when the experimental data are ambiguous functions, for example, when one value of the fuel consumption corresponds to several values of the gas-generator rotor r.p.m. The use of the linear models’ coefficients when determining the nonlinear model’s coefficients performs a regularization function. As a result of the sequential minimization of objective function (20), the nonlinear dynamic model coefficients are determined. Optimization tasks are solved for all of the experimental data values from the initial to the last with step h. It is advisable to minimize objective function (20) by using one of the direct unconditional optimization methods, for example, using the simplex method. Thus, as a result of the sequence solving the optimization tasks, all of the equations’ coefficient numerical values arrays that make up the helicopter TE model can be determined. It is advisable that the coefficient values of the helicopter TE dynamic model form functional dependencies on the parameter *α* using cubic spline interpolation or neural network approximation as they are necessary to calculate the injectivity processes using the identified model.

The helicopter TE nonlinear dynamic model identifying method was developed for the start-up and acceleration modes based on experimental data. The identification problem was formulated as a sequence of quadratic programming tasks where the objective functions served as local optimality criteria, enabling high accuracy in determining the model parameters. This approach leverages experimental data processing to ensure precise characterization of the engine’s dynamic behavior.

### 2.2. Development of the Helicopter Turboshaft Engine Neural Network Model

#### 2.2.1. Neural Network Architecture Development

For the helicopter TE dynamic multi-mode model to effectively solve the identifying task, it is necessary to analyze the existing control laws and improve them to ensure the optimal operating modes, which is impossible without adequate engine mathematical models in the form of differential equations. The model’s description is currently incomplete, since it does not take into account various connections and disturbances in the thermogas-dynamic processes occurring in the helicopter TE. In this regard, the most effective method is to use dynamic neural networks to approximate the results obtained from the research into thermogas-dynamic processes, for example, in a TV3-117 engine (Mi-8MTV helicopter) [71,72].

Currently, dynamic recurrent neural networks have been used to identify dynamic propagation objects, in particular, the nonlinear autoregressive network (e.g., NARX network) [73] and the Elman neural network, which is a special case of a multilayer recurrent network [74,75].

In [75], research was carried out to model the dynamic distribution of the TV3-117 engine’s operating process parameters. To assess the networks’ quantitative parameter modeling and the influence on accuracy, the NARX and Elman network parameters were used.

For the NARX network, the number of delay lines was changed to 1…5, with the number of hidden layer neurons being 1…20 using the Levenberg–Marquardt algorithm. The lowest mean square error (*MSE*) was 52.4 and was obtained for a network with 15 hidden layer neurons and three delay lines. However, the new data supplied to the input outside the training set indicate a discrepancy between the output signal and the required stability indicators. Therefore, the model did not meet the identification task.

For the Elman network, only the number of hidden layer neurons was varied, being 1…20, using the gradient descent algorithm with the training rate parameter perturbation and adaptation. The lowest *MSE* value of 41.83 was obtained for a single-layer network with two hidden layer neurons and double feedback delay. Increasing the number of hidden layers or delays led to a loss in the model sensitivity to changes in the input signals. Therefore, the best results were obtained on a single-layer network with a hidden layer and double feedback delay signal. The research results indicate that the model based on the Elman network was more stable compared to the NARX network and generally reflected the peculiarities of the dynamics of the helicopter TE parameters. Therefore, according to [75], the use of an Elman network with a hidden layer feedback signal double delay was proposed to simulate the dynamics of the helicopter TE parameters.

**Figure 5:**
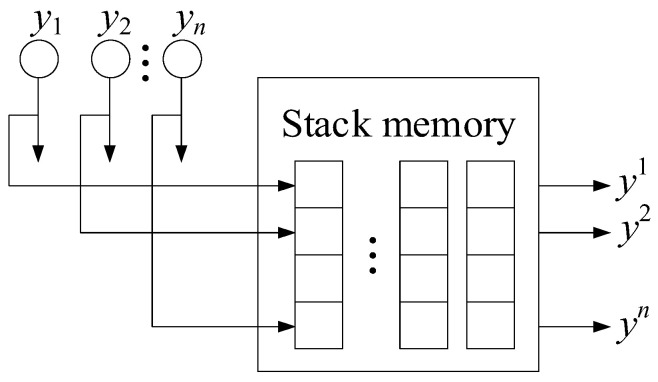
Implementation diagram of a memory layer for a modified Elman neural network with dynamic stack memory ([76], p. 134, URL: https://swsys.ru/index.php?page=article&id=3910&lang=.docs (accessed on 28 June 2024)).

For the Elman network training, gradient methods are used, similar to conventional feedforward networks with certain modifications [76,77]. This allows the error function gradient to be correctly calculated using the backpropagation method in time, turning the recurrent network into a “regular” one. The gradient (changes in weights) calculating process consists of a forward pass (the layers state calculating), a backward pass (the layers error determining), and calculating the change in weights based on the data from the previous stages. The adaptive storage of past temporal events is made possible by adding a dynamic memory stack (Figure 5) to recurrent neural networks, which allows it to provide and create a flexible tool for developing nonlinear models [76]. The modified Elman neural network is obtained by adding a delay to the hidden layer feedback signals (i.e., adding to the dynamic stack memory layer). 

Dynamic stack memory, when used in the Elman neural network, provides several significant advantages compared to alternative memory mechanisms such as recurrent mechanisms or fixed delays in recurrent networks. The primary advantage of dynamic stack memory lies in its ability to manage contextual information efficiently, offering flexibility in storing and retrieving data depending on the changing structure within the input data. Unlike traditional memory mechanisms, which are often limited by fixed memory volumes or rigid update rules, dynamic stack memory can adaptively alter its state, adjusting to current tasks, which is particularly beneficial for processing long data sequences with temporal dependencies. This property enhances network performance by retaining important information about past states more effectively for the accurate prediction of subsequent steps. The dynamic stack structure also allows for the reduction in redundant information, leading to decreased computational complexity compared to recurrent architectures, which often require the recalculation of all previous states at each iteration. As a result, dynamic stack memory not only reduces the computational load but also minimizes the loss of crucial information in complex tasks involving nonlinear dynamics, making it particularly attractive for real-time system applications such as helicopter TE control. 

A mathematical model of dynamic stack memory is given in Appendix A. Based on the above, the modified Elman neural network can be described without changes by recurrent equations [76]:

(21) $$v_{j}\left(t+1\right)=\sum_{i=1}^{N}w_{ji}^{\left(1\right)}\cdot u_{i}\left(t\right)+\sum_{i=1}^{T}w_{ji}^{\left(c\right)}\cdot x_{i}\left(t\right)+b_{j}^{\left(1\right)},\;x_{j}\left(t+1\right)=F_{1}\left(v_{j}\left(t+1\right)\right),\;y_{j}\left(t+1\right)=F_{2}\sum_{i=1}^{T}w_{ji}^{\left(2\right)}\cdot x_{i}\left(t+1\right)+b_{j}^{\left(2\right)},$$

or in matrix form:

**X**(*t* + 1) = **F**_1_(**W**^(1)^ · **U**(*t*) + **W**^(*c*)^ · **X**(*t*) + **B**^(1)^),

**Y**(*t* + 1) = **F**_2_(**W**^(2)^ · **X**(*t* + 1) + **B**^(2)^), (22)

where **U**(*t*) is the external input signal vector at time (*t*); *p* is the number of external network inputs; **X**(*t*) is the hidden layer output signal vector at time (*t*); *N* is the number of context layer signals; **W**^(1)^, **W***^c^*, **W**^(2)^ are the external input signals’ synaptic weight matrices, the context, and output layer signals, respectively; **B**^(1)^, **B**^(2)^ are the hidden and original layer neurons’ displacement weight vectors, respectively; **F**_1_, **F**_2_ are the hidden and original layers’ activation function vectors, respectively; **Y**(*t* + 1) is the network output signal vector at time (*t* + 1); *M* is the number of network outputs.

**Figure 6:**
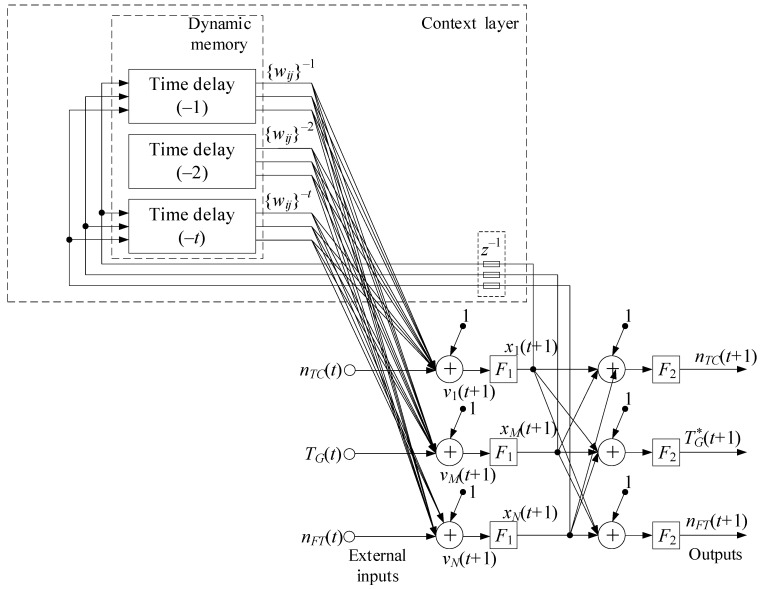
Modified Elman neural network with dynamic stack memory as a helicopter TE dynamic model (author’s research).

The hidden layer outputs, denoted as *v*_1_, *v*_2_, …, *v_N_*, are fed to the input neurons using weighting coefficients {*w_ij_*}^−*z*^, where *i* is the neuron index sending the signal (*i* = 1, 2, …, *t*); *j* is the hidden layer neuron output signal index (*j* = 1, 2, …, *k*); *z* = 1 is the time delay index (for the Elman network). A detailed examination of the neural network (Figure 6) architecture showed that feedback from the hidden layer or from the network output could be neglected by including feedback signals in the training set.

Furthermore, this architecture allows for the efficient training and adaptation of the network to the intricate dynamics of the engine’s operating modes. By incorporating the functional dependencies on the mode parameter *α*, the model ensures that the network can accurately map the engine’s states across its entire operational spectrum, from idle to emergency modes. This approach not only enhances the network’s predictive capabilities, but also ensures robustness and reliability in real-time applications, which are crucial for the helicopter TE’s optimal performance and safety.

Based on [76], for the modified Elman neural network with dynamic stack memory training, the training set was transformed. The training sample input signals, which change during the transformation, are designated as *x*_1_, *x*_2_, *x*_3_, and the feedback signals are *x*_4_, *x*_5_, *x*_6_, *x*_7_, *x*_8_, *x*_9_. The hidden layer output signals are supplied to inputs *x*_4_, *x*_5_, *x*_6_ with a one clock cycle delay *c*_1–1_, *c*_2–1_, *c*_3–1_; inputs *x*_7_, *x*_8_, *x*_9_ are the hidden layer output signals and are supplied with a two clock cycle delay *c*_1–2_, *c*_2–2_, *c*_3–2_. In this case, the layer memory is a layer output signal stack *y*_1_, *y*_2_, …, *y_n_*, where *n* is the stack size (see Figure 5). Each stack cell *y_i_* consists of the layer neuron outputs array *y*_1_, *y*_2_, …, *y_n_*. The stack is organized in such a way that after memory overflow, the last cell *y_n_* is deleted, and the entire queue is shifted by one position, so that *y_i_* = *y_i–1_*. The Elman network uses feedback between the hidden and input layers, implemented using a pure delay *z*^−1^ link. In the input layer, each hidden neuron has its own correspondence, which, together with the external input, forms the input information for the network. The initial layer contains neurons that the required engine parameter values calculate such as the gas-generator rotor r.p.m., the gas temperature in front of the compressor turbine, and the free turbine rotor speed.

Thus, the helicopter TE dynamic model, developed using a modified Elman neural network with dynamic stack memory, is closely linked to the physical relationships between the parameters recorded by on-board sensors. The gas-generator rotor r.p.m., measured by the D-2M sensor, reflects the primary rotor rotational speed responsible for driving the compressor and combustion process, directly influencing the air compression ratio and subsequent engine power output. The free turbine rotor speed, monitored by the D-1M sensor, measures the turbine rotational speed that is mechanically decoupled from the gas generator but drives the helicopter’s main rotor, providing thrust. This parameter is crucial for evaluating the energy transfer efficiency from the engine to the rotor system. The gas temperature in front of the compressor turbine, measured by a sensor comprising 14 thermocouples (T-101), is a key indicator of the thermal energy available to drive the turbine and is directly related to combustion efficiency and fuel consumption. Higher temperatures generally correlate with increased engine power output but also with potential thermal stress on engine components, necessitating careful monitoring. Fuel consumption, though not directly measured in the current configuration, influences all of other parameters, as the fuel combustion rate determines the energy produced, affecting rotor speeds and gas temperatures. Together, these parameters form a tightly coupled system that governs the helicopter TE’s performance and dynamic response. The dynamic model integrates these relations to predict engine behavior under various operational conditions, allowing for accurate real-time monitoring and control of the engine’s transitional states such as during starting, acceleration, and steady-state operations.

#### 2.2.2. Neural Network Training Algorithm Development

The training algorithm of the proposed modified Elman neural network with dynamic stack memory includes several steps such as data preparation, weight initialization, forward propagation, backpropagation, and weight updating.

During the data preparation stage, the training set’s input signals {*x*_1_, *x*_2_, *x*_3_} and feedback signals {*x*_4_, *x*_5_, *x*_6_, *x*_7_, *x*_8_, *x*_9_} are prepared, taking into account time delays. For an Elman network with dynamic stack memory, it is advisable to use the lagged features method, since it allows one to effectively take into account the time dependencies in the data. Lag functions create new features that represent the original feature values delayed by a certain number of time steps, which improves the network’s ability to train from a time series. This is especially important for recurrent neural networks, such as the Elman network, which uses information about previous states to predict future values. When using the lag function method to prepare data considering time delays, the analytical expressions for creating delayed features (lagged features with time delay *k*) look like:

*x*_1*lag*(*k*)_ = *x*_1_(*t* − *k*), *x*_2*lag*(*k*)_ = *x*_2_(*t* − *k*), *x*_3*lag*(*k*)_ = *x*_3_(*t* − *k*), *x*_4*lag*(*k*)_ = *x*_4_(*t* − *k*), *x*_5*lag*(*k*)_ = *x*_5_(*t* − *k*), *x*_6*lag*(*k*)_ = *x*_6_(*t* − *k*), *x*_7*lag*(*k*)_ = *x*_7_(*t* − *k*),

*x*_8*lag*(*k*)_ = *x*_8_(*t* − *k*), *x*_9*lag*(*k*)_ = *x*_9_(*t* − *k*), (23)

where *k* is any integer representing the time delay, and (*t* − *k*) represents the feature *x* value at time step (*t* − *k*). Thus, each feature from the original data is expanded to include its values at different delays, which allows us to take into account the dynamics and time dependencies in the data.

To improve the stability and accuracy of the optimization algorithm, filtering can be added by using an *n*-th order Butterworth filter, that is:

*x*_1*lag*(*k*)_ = ButterworthFilter(*x*_1*lag*(*k*)_, *k*), *x*_2*lag*(*k*)_ = ButterworthFilter(*x*_2*lag*(*k*)_, *k*), *x*_3*lag*(*k*)_ = ButterworthFilter(*x*_3*lag*(*k*),_ *k*),

*x*_4*lag*(*k*)_ = ButterworthFilter(*x*_4*lag*(*k*)_, *k*), *x*_5*lag*(*k*)_ = ButterworthFilter(*x*_5*lag*(*k*)_, *k*), *x*_6*lag*(*k*)_ = ButterworthFilter(*x*_6*lag*(*k*)_, *k*),

*x*_7*lag*(*k*)_ = ButterworthFilter(*x*_7*lag*(*k*)_, *k*), *x*_8*lag*(*k*)_ = ButterworthFilter(*x*_8*lag*(*k*)_, *k*), *x*_9*lag*(*k*)_ = ButterworthFilter(*x*_9*lag*(*k*)_, *k*), (24)

where ButterworthFilter represents the delayed features (lagged features with time delay *k*) filtering function *x*_1_*_lag_*_(_*_k_*_)_…*x*_9_*_lag_*_(_*_k_*_)_ at time delay *k*.

Thus, the input signal *x_i_*(*t*) passes the *n*-th order Butterworth filter specified by the transfer function:

(25) $$H\left(s\right)=\frac{1}{1+{\left(\frac{s}{\omega_{c}}\right)}^{2\cdot n}},$$

where *ω_c_* is the filter cutoff frequency, and *s* is the complex Laplace variable.

For discrete time *t* = 1, 2, …, *T*, the filtered signal $x_{i}^{BF}\left(t\right)$ is defined as:

(26) $$x_{i}^{BF}\left(t\right)=F^{-1}\left\{H\left(s\right)\cdot F\left(x_{i}\left(t\right)\right)\right\},$$

where F denotes the Fourier transform operator, and $F^{-1}$ is the inverse Fourier transform.

After preparing the input data, the weights and parameters of Adam optimization algorithm are initialized as:

(27) $$W^{\left(1\right)}=\mathrm{rand}(N,p),W^{\left(c\right)}=\mathrm{rand}(N,N),W^{\left(2\right)}=\mathrm{rand}(M,N),B^{\left(1\right)}=\mathrm{rand}(N,1),B^{\left(2\right)}=\mathrm{rand}(M,1),\;m_{W^{\left(1\right)}}=0,\;v_{W^{\left(1\right)}}=0,\;m_{W^{\left(c\right)}}=0,\;v_{W^{\left(c\right)}}=0,\;m_{W^{\left(2\right)}}=0,\;v_{W^{\left(2\right)}}=0,\;m_{B^{\left(1\right)}}=0,\;v_{B^{\left(1\right)}}=0,\;m_{B^{\left(2\right)}}=0,\;v_{B^{\left(2\right)}}=0,$$

in this case, *β*_1_ = 0.9, *β*_2_ = 0.999, *ϵ* = 10^−8^ are the Adam algorithm parameters. Parameter *β*_1_ is the gradient first moment exponential decay rate. The *β*_1_ = 0.9 value means that the current value of the first moment (the gradient first moment estimate) is obtained from the previous value of the first moment with a weight of 0.9 and the current gradient with a weight of 0.1. Parameter *β*_2_ is the gradient second moment exponential moving average. The *β*_2_ = 0.999 value means that the current value of the second moment (the gradient second moment estimate) is obtained from the previous value of the second moment with weight of 0.999 and the current gradient square with a weight of 0.001. The parameter *ϵ* is a small number added for numerical stability and prevents division by zero in the training step adjustment formula. The value *ϵ* = 10^−8^ is standard, and is used to avoid possible numerical tasks with division.

After initializing the weights and parameters of the Adam optimization algorithm, direct propagation is carried out, in which at each time step *t*, the output signals of the hidden and output layers are calculated according to Equation (22), that is:

*X*(*t* + 1) = *F*_1_(*W*^(1)^ · *U*(*t*) + *W*^(*c*)^ · *X*(*t*) + *B*^(1)^),

*Y*(*t* + 1) = *F*_2_(*W*^(2)^ · *X*(*t* + 1) + *B*^(2)^). (28)

The next step (backpropagation step) calculates the error at the output layer as the difference between the target values and the actual output signals:

*E*(*t* + 1) = *D*(*t* + 1) − *Y*(*t* + 1), (29)

where *D*(*t* + 1) – the target values at step (*t* + 1).

The weights of the output layer are updated according to the expression:

(30) $$\delta_{h}={\left(W^{\left(2\right)}\right)}^{T}\cdot E\left(n+1\right)⊙\left(F_{1}^{\prime }X\left(n+1\right)\right),$$

where ⊙ denotes element-wise multiplication.

Gradients are calculated as follows:

(31) $$g_{W^{\left(1\right)}}=E\left(n+1\right)\cdot \left(F_{2}^{\prime }Y\left(n+1\right)\right)\cdot {\left(X\left(n+1\right)\right)}^{T},g_{W^{\left(2\right)}}=\delta_{h}\cdot {\left(U\left(n\right)\right)}^{T},g_{B^{\left(1\right)}}=\delta_{h},g_{B^{\left(1\right)}}=E\left(n+1\right).$$

The parameters of the Adam optimization algorithm are updated as follows:

(32) $$m_{W^{\left(1\right)}}=\beta_{1}\cdot m_{W^{\left(1\right)}}+\left(1-\beta_{1}\right)\cdot g_{W^{\left(1\right)}},\;v_{W^{\left(1\right)}}=\beta_{2}\cdot v_{W^{\left(1\right)}}+\left(1-\beta_{2}\right)\cdot \left(g_{W^{\left(1\right)}}⊙g_{W^{\left(1\right)}}\right),\;m_{W^{\left(c\right)}}=\beta_{1}\cdot m_{W^{\left(c\right)}}+\left(1-\beta_{1}\right)\cdot g_{W^{\left(c\right)}},\;v_{W^{\left(c\right)}}=\beta_{2}\cdot v_{W^{\left(c\right)}}+\left(1-\beta_{2}\right)\cdot \left(g_{W^{\left(c\right)}}⊙g_{W^{\left(c\right)}}\right),\;m_{W^{\left(2\right)}}=\beta_{1}\cdot m_{W^{\left(2\right)}}+\left(1-\beta_{1}\right)\cdot g_{W^{\left(2\right)}},\;v_{W^{\left(2\right)}}=\beta_{2}\cdot v_{W^{\left(2\right)}}+\left(1-\beta_{2}\right)\cdot \left(g_{W^{\left(2\right)}}⊙g_{W^{\left(2\right)}}\right),\;m_{B^{\left(1\right)}}=\beta_{1}\cdot m_{B^{\left(1\right)}}+\left(1-\beta_{1}\right)\cdot g_{B^{\left(1\right)}},\;v_{B^{\left(1\right)}}=\beta_{2}\cdot v_{B^{\left(1\right)}}+\left(1-\beta_{2}\right)\cdot \left(g_{B^{\left(1\right)}}⊙g_{B^{\left(1\right)}}\right),\;m_{B^{\left(2\right)}}=\beta_{1}\cdot m_{B^{\left(2\right)}}+\left(1-\beta_{1}\right)\cdot g_{B^{\left(2\right)}},\;v_{B^{\left(2\right)}}=\beta_{2}\cdot v_{B^{\left(2\right)}}+\left(1-\beta_{2}\right)\cdot \left(g_{B^{\left(2\right)}}⊙g_{B^{\left(2\right)}}\right).$$

The correction of the parameters of the Adam optimization algorithm is carried out as follows:

(33) $${\hat{m}}_{W^{\left(1\right)}}=\frac{m_{W^{\left(1\right)}}}{1-\beta_{1}^{t}},\;{\hat{v}}_{W^{\left(1\right)}}=\frac{v_{W^{\left(1\right)}}}{1-\beta_{2}^{t}},{\hat{m}}_{W^{\left(c\right)}}=\frac{m_{W^{\left(c\right)}}}{1-\beta_{1}^{t}},\;{\hat{v}}_{W^{\left(c\right)}}=\frac{v_{W^{\left(c\right)}}}{1-\beta_{2}^{t}},\;{\hat{m}}_{W^{\left(2\right)}}=\frac{m_{W^{\left(2\right)}}}{1-\beta_{1}^{t}},\;{\hat{v}}_{W^{\left(2\right)}}=\frac{v_{W^{\left(2\right)}}}{1-\beta_{2}^{t}},\;{\hat{m}}_{B^{\left(1\right)}}=\frac{m_{B^{\left(1\right)}}}{1-\beta_{1}^{t}},\;{\hat{v}}_{B^{\left(1\right)}}=\frac{v_{B^{\left(1\right)}}}{1-\beta_{2}^{t}},\;{\hat{m}}_{B^{\left(2\right)}}=\frac{m_{B^{\left(2\right)}}}{1-\beta_{1}^{t}},\;{\hat{v}}_{B^{\left(2\right)}}=\frac{v_{B^{\left(2\right)}}}{1-\beta_{2}^{t}}.$$

Updating weights and biases is conducted as follows:

(34) $$W^{\left(1\right)}\leftarrow W^{\left(1\right)}-\alpha \cdot \frac{{\hat{m}}_{W^{\left(1\right)}}}{\sqrt{\;{\hat{v}}_{W^{\left(1\right)}}}}+\epsilon ,\;W^{\left(c\right)}\leftarrow W^{\left(c\right)}-\alpha \cdot \frac{{\hat{m}}_{W^{\left(c\right)}}}{\sqrt{\;{\hat{v}}_{W^{\left(c\right)}}}}+\epsilon ,W^{\left(2\right)}\leftarrow W^{\left(2\right)}-\alpha \cdot \frac{{\hat{m}}_{W^{\left(2\right)}}}{\sqrt{\;{\hat{v}}_{W^{\left(2\right)}}}}+\epsilon ,\;B^{\left(1\right)}\leftarrow B^{\left(1\right)}-\alpha \cdot \frac{{\hat{m}}_{B^{\left(1\right)}}}{\sqrt{\;{\hat{v}}_{B^{\left(1\right)}}}}+\epsilon ,\;B^{\left(2\right)}\leftarrow B^{\left(2\right)}-\alpha \cdot \frac{{\hat{m}}_{B^{\left(2\right)}}}{\sqrt{\;{\hat{v}}_{B^{\left(2\right)}}}}+\epsilon ,$$

where *α* is the initial training parameter.

During stack memory processing, after a memory overflow, the last cell *y_n_* is deleted, and the entire queue is shifted by one position, that is:

*y_i_* = *y_i_*_–1_ (35)

for all *i* ∈ [2, *t*].

Thus, the proposed algorithm using signal filtering using the *n*-th order Butterworth filter and Adam adaptive training coefficient provides the modified Elman neural network with more stable and faster training.

The scientific novelty of the proposed algorithm lies in the signal filtering integration using an *n*-th order Butterworth filter and an adaptive training coefficient based on the Adam method into a modified Elman neural network with dynamic stack memory. This approach significantly improves the training process by automatically adapting the training rate to changes in the network parameter gradients during training, thereby achieving the optimal weights and biases more efficiently. Unlike the traditional Elman network training algorithm, which uses a static training rate, the new method takes into account dynamic changes in the data and network structure, which increases its adaptability and ability to effectively optimize under the variety of input and feedback signals.

#### 2.2.3. Description of the Method for the Neural Network Training Sample Generating, Analyzing, and Preprocessing

The training sample consisted of helicopter TE parameters *n_TC_*(*t*), *n_FT_*(*t*), and $T_{G}^{*}$(*t*), recorded on-board the helicopter under flight operating conditions in the time interval *T* with a sampling period Δ*t*. To prepare the data for the modified Elman neural network training with dynamic stack memory, the training sample was transformed, as a result of which a transformed training sample was formed. To determine its possibility for use in training a modified Elman neural network with dynamic stack memory, its homogeneity and representativeness were assessed [77,78,79].

The transformed training sample homogeneity was assessed using the Fisher–Pearson criterion [80], defined as:

(36) $$\chi^{2}=\sum_{i}\frac{{\left(O_{i}-E_{i}\right)}^{2}}{E_{i}},$$

where *O_i_* is the observed frequency in the *i*-th category (the sample observed *i*-th values are the actual frequencies), and *E_i_* is the expected frequency in the *i*-th category (the frequencies that are expected in each category if the uniform distribution hypothesis is true).

The *χ*^2^ statistic has a chi-square distribution with (*k* − 1) degrees of freedom, where *k* is the number of categories. To check the sample homogeneity, the chi-square test is used: if the obtained value of the *χ*^2^ statistic is greater than the critical value from the chi-square distribution table for a given significance level and number of degrees of freedom, then the homogeneity hypothesis is rejected.

To confirm the homogeneity assessment of the transformed training set, the Fisher–Snedecor test (*F*-test) [81] was used, which is used to assess the variances in homogeneity between several groups or samples. In the assessing context of the training set homogeneity, it can be used to compare the data with different group variances. The Fisher–Snedecor criterion is defined as:

(37) $$F=\frac{MSB}{MSE},$$

where *MSB* (mean square between) is the mean square error between groups, calculated as $MSB=\frac{\sum_{i=1}^{k}n_{i}\cdot {\left({\bar{X}}_{i}-\bar{X}\right)}^{2}}{k-1}$, where ${\bar{X}}_{i}$ is the average value in the *i*-th group, $\bar{X}$ is the overall average for all groups, *k* is the number of groups with data, *n_i_* is the number of observations in the *i*-th group; *MSE* (mean square error) is the mean square error within groups, calculated as $MSE=\frac{\sum_{i=1}^{k}S_{i}\cdot \left(n_{i}-1\right)}{N-k}$, where *S_i_* is the sample variance in the *i*-th group, and *N* is the total number of observations.

The *F* statistic has a Fisher distribution with parameters (*k* − 1) and (*N* − *k*) degrees of freedom. If the obtained value of the *F* statistic is greater than the critical value from the *F* distribution table for a given significance level and the number of degrees of freedom, then the homogeneity hypothesis is rejected.

Based on [77,78,79], to assess the representativeness of the training sample, cluster analysis was used in the form of the *k*-means method, which is based on the parameters of intracluster and intercluster distance [82,83,84,85].

The average within-cluster distance shows how similar objects are within the same cluster. The smaller the distance, the more compact the cluster, which may indicate training data with a more representative selection for that cluster. The average intracluster distance is defined as:

(38) $$W=\sum_{i=1}^{k}\frac{1}{n_{i}}\cdot \sum_{x\in C_{i}}{\left\|x-\mu_{i}\right\|}^{2},$$

where *k* is the number of clusters, *n_i_* is the number of cluster *C_i_* points, *μ_i_* is the cluster *C_i_* centroid, and ${\left\|x-\mu_{i}\right\|}^{2}$ is the squared Euclidean distance between point *x* and centroid *μ_i_*.

The intercluster distance shows the distance between the centroids of the different clusters. The larger the distance, the more distinct and separated the clusters are from each other, which may indicate a more representative selection of the training data. The intercluster distance for *k*-means is defined as:

(39) $$B=\sum_{i=1}^{k}n_{i}\cdot {\left\|\mu_{i}-\mu \right\|}^{2},$$

where *μ* is the common centroid for all points.

Calculations according to Equations (38) and/or (39) continue until the centroids cease to change significantly. The process stops when a convergence criterion is reached, for example, when changes in the centroids become insignificant and can be neglected. During the execution of the *k*-means algorithm, it seeks to minimize the sum of the squared distances from each data point to its cluster centroid, that is:

(40) $$J=\sum_{i=1}^{k}{\left\|x_{i}-\mu_{i}\right\|}^{2}.$$

#### 2.2.4. Description of the Computational Experiment Conducting Algorithm

The basis of the computational experiment was the proposed Elman neural network with dynamic stack memory training. A training curve was used to determine the optimal number of epochs for training. The basic idea is to find the point at which the error on the validation set is minimized or begins to stabilize, indicating that improvements will cease with further training. For each epoch *t* (from 1 to *T*), the mean square error (*MSE*) is calculated on the validation set *MSE_val_*(*t*). The optimal number of epochs *t*^∗^ is defined as:

(41) $$t^{*}=min\left\{t|\forall t^{\prime },\left|{MSE}_{val}\left(t^{\prime }\right)-{MSE}_{val}\left(t^{\prime }\right)\left|<\epsilon \right.\right.\right\}.$$

This means that *t*^∗^ is the first epoch from which the change in error on the validation set is less than the threshold value *ϵ* for all subsequent epochs.

Additionally, key indicators of the quality of the neural network training are *Training Accuracy* and *Validation Accuracy*. The *Training Accuracy* versus *Validation Accuracy* diagrams showed a change in accuracy on the training and validation data depending on the training epoch. In this case, the accuracy metric is calculated as:

(42) $$Accuracy=\frac{1}{N}\cdot \sum_{i=1}^{N}1\left(y_{i}={\hat{y}}_{i}\right),$$

where *y_i_* is the *i*-th example of the true label, ${\hat{y}}_{i}$ is the *i*-th example label predicted by the model, *N* is the number of examples in the dataset (training or validation), and $1\left(y_{i}={\hat{y}}_{i}\right)$ is the indicator function, which is equal to 1 if $y_{i}={\hat{y}}_{i}$, and 0 otherwise.

An important indicator of the training quality of the neural network is the training error (*Training Loss*) and validation error (*Validation Loss*). To develop training error (*Training Loss*) and validation error (*Validation Loss*) diagrams, it is necessary to calculate the loss function values (the *MSE* is taken) on the training and validation data at each training epoch. In this case, the *MSE* is calculated as:

(43) $$MSE=\frac{1}{N}\cdot \sum_{i=1}^{N}{\left(y_{i}-{\hat{y}}_{i}\right)}^{2}.$$

To develop a gradient diagram (gradient norms), which shows the gradient norms at each training epoch, it is necessary to calculate the gradients norms of the model parameters at each epoch. The gradient norm allows for changes to be monitored in the gradients and problems to be identified such as disappearing or exploding gradients. This work adopted the gradient L2-norm (*Euclidean norm*), which is calculated as:

(44) $${\left\|\nabla L\left(\theta \right)\right\|}_{2}=\sqrt{\sum_{i}{\left(\frac{\partial L}{\partial \theta_{i}}\right)}^{2}},$$

where *θ* is the vector for all model parameters, ∇*L*(*θ*) is the loss function *L* gradient vector over *θ_i_* parameters. The loss function gradient calculating mathematical model is given in Appendix B.

Validation of the training results of the proposed Elman neural network with dynamic stack memory was carried out using cross-validation [85], in which the data are divided into several parts (folds), and the model is trained and evaluated several times, each time on data different parts.

To carry out cross-validation (k-fold cross-validation), the training sample data are divided into k early parts (folds): *D*_1_, *D*_2_, …, *D_k_*. For each *i*-th fold (*i* = 1, 2, …, *k*), one part of the data is used as a validation set, and the remaining *k* − 1 folds are used for training. The model is trained on *k* − 1 data folds and evaluated on the remaining one data fold. For each *i*-th iteration, the model is trained on data $D_{train}^{\left(i\right)}$ and evaluated on data $D_{val}^{\left(i\right)}$, where:

(45) $$D_{train}^{\left(i\right)}=\underset{j\neq i}{⋃}D_{j},\;D_{val}^{\left(i\right)}=D_{i}.$$

Next, the metric is calculated (in this work, the *MSE* was taken as this metric) on the validation sample for each *i*-th fold:

(46) $${Metric}_{i}=evalute\left(M^{\left(i\right)},D_{val}^{\left(i\right)}\right),$$

where *M*^(*i*)^ is a model trained on $D_{val}^{\left(i\right)}$.

After completing all *k* iterations, the average metric value is calculated over all folds:

(47) $$Average\;Metric=\frac{1}{k}\cdot \sum_{i=1}^{k}{Metric}_{i}.$$

#### 2.2.5. Description of the Computational Experiment Conducting Algorithm

To assess the quality of the computational experiment conducted using a modified Elman neural network with dynamic stack memory to identify the helicopter TE dynamic model, traditional statistical metrics were used in the research [86].

The applied metrics serve as the neural networks’ functioning accuracy and stability quantitative measures, providing researchers and developers with a tool for the comparative analysis of various algorithms and architectures.

The determination coefficient (*R*^2^) in the helicopter TE dynamic model identifying context shows that the dependent variable’s (for example, the helicopter TE parameters or characteristics) variance proportion can be explained by the model used, reflecting its suitability for predicting and analyzing flight data. The determination coefficient is defined as:

(48) $$R^{2}=1-\frac{{\sum_{i=1}^{N}\left(y_{i}-{\hat{y}}_{i}\right)}^{2}}{{\sum_{i=1}^{N}\left(y_{i}-\bar{y}\right)}^{2}},$$

where *y_i_* is the real value, ${\hat{y}}_{i}$ is the predicted (identified) value, $\bar{y}$ is the average value of the real value *y_i_*.

The determination coefficient, adjusted for the number of parameters (adjusted R-squared), taking into account the number of predicted variables, helps to avoid overfitting and more accurately assess the model quality. The *R*^2^ adjusted version is defined as:

(49) $$R_{adj}^{2}=1-\frac{\left(1-R^{2}\right)\cdot \left(N-1\right)}{N-K-1},$$

where *N* is the number of observations and *K* is the number of predicted variables.

The mean absolute error (*MAE*) in the helicopter TE dynamic model identification context shows the average absolute deviation between the helicopter TE parameters’ actual and predicted values, which helps to assess the accuracy of the model. The mean absolute error is defined as:

(50) $$R^{2}=\frac{1}{N}\cdot \sum_{i=1}^{N}\left|y_{i}-{\hat{y}}_{i}\right|.$$

The correlation coefficient shows the degree of linear dependence between the helicopter TE parameters’ real and predicted (identified) values. The correlation coefficient is defined as:

(51) $$r=\frac{\sum_{i=1}^{N}\left(y_{i}-\bar{y}\right)\cdot \left({\hat{y}}_{i}-\bar{\hat{y}}\right)}{\sqrt{\sum_{i=1}^{N}{\left(y_{i}-\bar{y}\right)}^{2}\cdot \sum_{i=1}^{N}{\left({\hat{y}}_{i}-\bar{\hat{y}}\right)}^{2}}},$$

where $\bar{\hat{y}}$ is the predicted (identified) value and ${\hat{y}}_{i}$ is the average value.

*Relative error* expresses the average relative deviation between the actual and predicted (identified) values, which is useful for assessing the model percentage accuracy. The relative error is defined as:

(52) $$RE=\frac{1}{N}\cdot \sum_{i=1}^{N}\left|\frac{y_{i}-{\hat{y}}_{i}}{y_{i}}\right|\times 100\%.$$

*Foote’s metric* evaluates the neural network quality by its ability to identify the main components of the data, which is especially important for complex and dynamic models such as helicopter TEs. *Foote’s metric* is defined as:

(53) $$FM=\frac{\sum_{i=1}^{N}{\left({\hat{y}}_{i}-\bar{\hat{y}}\right)}^{2}}{{\sum_{i=1}^{N}\left(y_{i}-{\hat{y}}_{i}\right)}^{2}}.$$

This work also used the metrics *Accuracy*, *Precision*, *Recall*, *F1-score*, which are standard tools for assessing the predictive models’ quality in the helicopter TE dynamic model identifying context, taking into account both the predictions’ completeness and accuracy, which is especially important for models used for flight operation.

*Accuracy*, in the helicopter TE dynamic model identifying context, shows the proportion of correctly classified observations (helicopter TE correctly identified parameters or characteristics) from the total number of observations, which reflects the model’s overall accuracy. *Accuracy* is defined as [87,88]:

(54) $$Accuracy=\frac{TP+TN}{TP+TN+FP+FN},$$

where *TP* (True Positive) is the correctly identified positive helicopter TE parameters, *TN* (True Negative) is the correctly identified negative helicopter TE parameters, *FP* (False Positive) is the falsely identified positive helicopter TE parameters, and *FN* (False Negative) is the falsely identified negative helicopter TE parameters.

*Precision*, in the helicopter TE dynamic model identifying context, shows the correctly identified positive cases (for example, the helicopter TE certain features) among the proportion of all of the model’s positive predictions. *Precision* is defined as:

(55) $$Precision=\frac{TP}{TP+FP}.$$

*Recall*, in the helicopter TE dynamic model identifying context, shows the correctly identified positive cases (for example, the helicopter TE operation defects or deviations) among the proportion of all real positive cases, which reflects the model’s ability to detect all existing positive cases. *Recall* is defined as:

(56) $$Recall=\frac{TP}{TP+FN}.$$

*F1-score* in the helicopter TE dynamic model identification context is the harmonic average between *Precision* and *Recall*, providing a balanced assessment of the model’s accuracy in detecting and identifying positive cases. *F1-score* is defined as:

(57) $$F1-score=\frac{2\cdot Precision\cdot Recall}{Precision+Recall}.$$

The parameters *TP*, *TN*, *FP*, *FN* represent an error matrix. The error matrix (confusion matrix) in the helicopter TE dynamic model identifying context is a table that shows the number of correctly and incorrectly classified observations for each class. In the case of binary classification for the helicopter TE model, the error matrix has the form presented in Table 1.

It is noted that the *TN* parameter is missing in the general error matrix. In classification tasks, where class includes the helicopter TE dynamic model identifying task, with more than two classes, the TN parameter is interpreted a little differently. In the standard error matrix for multi-class tasks, there are many *TN* values for each observation, which makes accounting for them more difficult. Thus, *TN* denotes the number of cases where neither the transient nor the transient is classified as stationary (correct non-recognition).

This work performed ROC analysis to evaluate the model’s ability to accurately distinguish between classes (for example, the helicopter TE normal operation and defects), allowing the trade-off between sensitivity analysis (True Positive Rate) and specificity (1 − False Positive Rate) at different classification thresholds.

An *ROC* curve was constructed by varying the model’s classification threshold and measuring its ability to discriminate between classes. This is the True Positive Rate (*TPR*) versus False Positive Rate (*FPR*) diagram.

The True Positive Rate (*TPR*) is the correctly classified positive proportion of the true positive cases with the total number of cases out and is defined as:

(58) $$TPR=\frac{TP}{TP+FN}.$$

The False Positive Rate (*FPR*) is the total number of the true negative cases incorrectly classified out of the proportion of negative cases and is defined as:

(59) $$FPR=\frac{FP}{FP+TN}.$$

The *AUC-ROC* is a numeric value that represents the probability that the model correctly classifies a randomly selected positive example that is higher than a randomly selected negative example. The area under the *ROC* curve is defined as:

(60) $$AUC-ROC=\int_{0}^{1}TPR\cdot \left({FPR}^{-1}\left(t\right)\right)dt,$$

where *TPR* (True Positive Rate) is the sensitivity, *FPR*^−1^ is the False Positive Rate (1 is specificity) inverse function, and *t* is the classification threshold value, varying from 0 to 1.

This work also involved first and second type error calculations, which in the helicopter TE dynamic model identifying in a flight operation task context is necessary to assess the accuracy and reliability of the engine condition monitoring and prediction system, which is critically important for ensuring flight safety and operational efficiency.

A first type error (omission error) occurs when the system does not detect the presence of an event or malfunction that has actually occurred. In the helicopter TE model identifying context, this may mean that the system did not recognize or incorrectly recognized the engine’s current state or parameters. A first type error is defined as:

(61) $$\alpha =P\left(reject\;H_{0}\left|H_{0}\right.\;true\right).$$

A second type error (false alarm) occurs when the system falsely reports the event or there is the presence of a fault that is not actually present. In this task, this can manifest itself as a false positive in the model identification system when it mistakenly identifies a fault where there is none. A second type error is defined as:

(62) $$\beta =P\left(\mathrm{do}\mathrm{not}\mathrm{reject}\;H_{0}\left|H_{0}\right.\;false\right)=1-\beta .$$

Moreover, in the helicopter TE dynamic model identifying task context, the null hypothesis *H*_0_ is presented as follows: “There is no statistically significant difference between the helicopter TE model predicted and real parameters obtained using a modified Elman neural network with dynamic stack memory”.

At the same time, an alternative hypothesis was accordingly formulated as: “There is a statistically significant difference between the helicopter TE model predicted and real parameters, obtained using a modified Elman neural network with dynamic stack memory, confirming the dynamic model identifying effectiveness at helicopter flight conditions”.

It is noted that in the helicopter TE dynamic model identifying task context, setting the significance level to 0.01 is justified by the need to ensure the high reliability of the identification results. This means that the null hypothesis falsely rejecting probability, if true, is only 1%. For the operational and safe operation of helicopters, it is critical to the engine model identification system to minimize false likelihood alarms in order to prevent unforeseen situations and ensure system stability at real operating conditions. This significance level provides more accurate and reliable results, which is especially important in the aerospace industry, where any error can have serious consequences for flight safety.

Thus, the application of clearly defined Formulas (44)–(58) and their use in the article contribute to ensuring the reproducibility and verification of the research results, which is the foundation for further development in the field of artificial intelligence and machine learning.

## 3. Case Study

### 3.1. The Input Data Analysis and Pre-Processing Results

**Figure 7:**
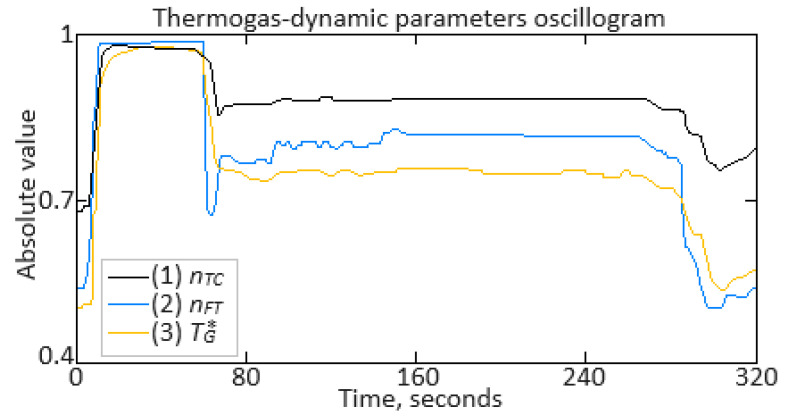
The TV3-117 turboshaft engine parameters’ dynamics time series using digitized oscillograms. (**Black curve**) Gas-generator rotor r.p.m; (**Blue curve**) free turbine rotor speed; (**Orange curve**) gas temperature in front of the compressor turbine (author’s research).

To carry out the computational experiment, the data were recorded during the TV3-117 TE on-board the Mi-8MTV helicopter flight tests using an on-board control system (data recording occurred in 320 s intervals from a real flight with a 1.0 s sampling period [73]). It is noted that the flight data were obtained based on the official request of the authors’ collective by the Ministry of Internal Affairs of Ukraine within the framework of the research project “Theoretical and applied aspects of the development of the aviation sphere” with state registration in Ukraine (No. 0123U104884). Based on the data obtained, time series diagram was reconstructed with the parameters *n_TC_*, *n_FT_*, and $T_{G}^{*}$ (recorded on-board the helicopter using sensors D-2M, D-1M, and 14 T-101 dual thermocouples, respectively) (Figure 7). The dynamics of the TV3-117 TE parameters reflects the complexity of the shape of the time series (Figure 7), and the very appearance of curves indicates the need to take into account the parameter values and accumulate information in the model’s memory.

Figure 7 shows an increase in the parameter values in the time interval from 20 to 65 s by approximately 1.5…1.8 times, which is explained by the engine operating in transient mode. As stated in the introduction, about 85% of the time, the engine operates in steady-state conditions, and only about 15% in transient conditions. Based on [73], 256 of the *n_TC_*, *n_FT_*, and $T_{G}^{*}$ values were selected according to Figure 7 (Table 2). This training sample size was justified by ensuring sufficient coverage of the possible variations in the helicopter TE parameters, allowing the modified Elman neural network with dynamic stack memory to effectively train and accurately identify the engine dynamic model in flight operating conditions. In addition, a sample of 256 values was sufficient to ensure the normal distribution condition, which is critical for the statistical significance and reliability of the training set in the neural network using context to identify the helicopter TE dynamic model.

To determine the homogeneity of the training sample, according to Equation (36), the value *χ*^2^ = 24.935 was determined, which was less than the critical value $\chi_{critical}^{2}=27.683$ for *df* = 13 degrees of freedom at an α = 0.01 significance level. The results obtained indicate the training sample homogeneity according to the Fisher–Pearson criterion. To confirm homogeneity, the *N* = 256 training sample size was divided into two subsamples of the same size: *n*_1_ = *n*_2_ = 128 elements. According to Equation (37), the value *F* = 5.58 was obtained, which was less than the critical value *F_critical_* = 5.74. The results obtained confirm the homogeneity of the training set according to the Fisher–Snedecor criterion.

**Figure 8:**
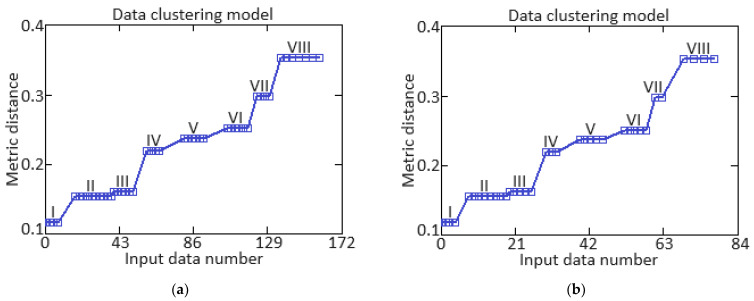
Cluster analysis results: (**a**) training set, (**b**) test set (author’s research).

Based on the training sample of the helicopter TE thermogas-dynamic parameters (Table 2), a random selection procedure was used to select training and test samples in a 2:1 ratio (67 and 33%, which are 172 and 84 elements, respectively). The data cluster analysis results from the training sample of the helicopter TE thermogas-dynamic parameters (Table 2) revealed eight classes (classes I…VIII), that is, the presence of eight groups within them, which indicate the similarity in composition of both the training and test samples (Figure 8).

The results obtained made it possible to determine the optimal sample size for the helicopter TE thermogas-dynamic parameters: a training sample of 256 elements (100%), control sample of 172 elements (the training sample 67%), and test sample of 84 elements (training sample of 33%).

It was noted that the curves of the helicopter TE parameters (Figure 7) contained abrupt changes in their values, which made it necessary to perform preliminary low-frequency filtering on the experimental data [57,63]. Therefore, at the pre-processing stage of the training sample of the helicopter TE thermogas-dynamic parameters (Table 2), in addition to compensating for the shortcomings of on-board recording system, the source data, as a rule, required high-frequency interference filtering from the information and measuring channels.

As described earlier, the use of a Butterworth filter has been proposed, which belongs to the class of filters with an infinite impulse response. In this case, the Butterworth filter has a filtered signal with a significant phase shift peculiarity. To eliminate this, the filtering procedure is carried out twice: first in the forward direction, and then in the reverse direction [63,64].

A description of the Butterworth filter is given in the form of row vectors *b* and *a*, which have the length *n* + 1 and contain the transfer function numerator and denominator polynomial coefficients in the variable *z* powers descending [55,56]:

(63) $$H\left(z\right)=\frac{B\left(z\right)}{A\left(z\right)}=\frac{b_{1}+b_{2}\cdot z^{-1}+\ldots +b_{n+1}\cdot z^{-n}}{1+a_{2}\cdot z^{-1}+\ldots +a_{n+1}\cdot z^{-n}}.$$

It is noted that the choice of the cutoff frequency *ω_c_* depends on the obtained spectral characteristics. The filter order *n* affects the characteristic flatness after the cutoff frequency *ω_c_* [63,64].

**Figure 9:**
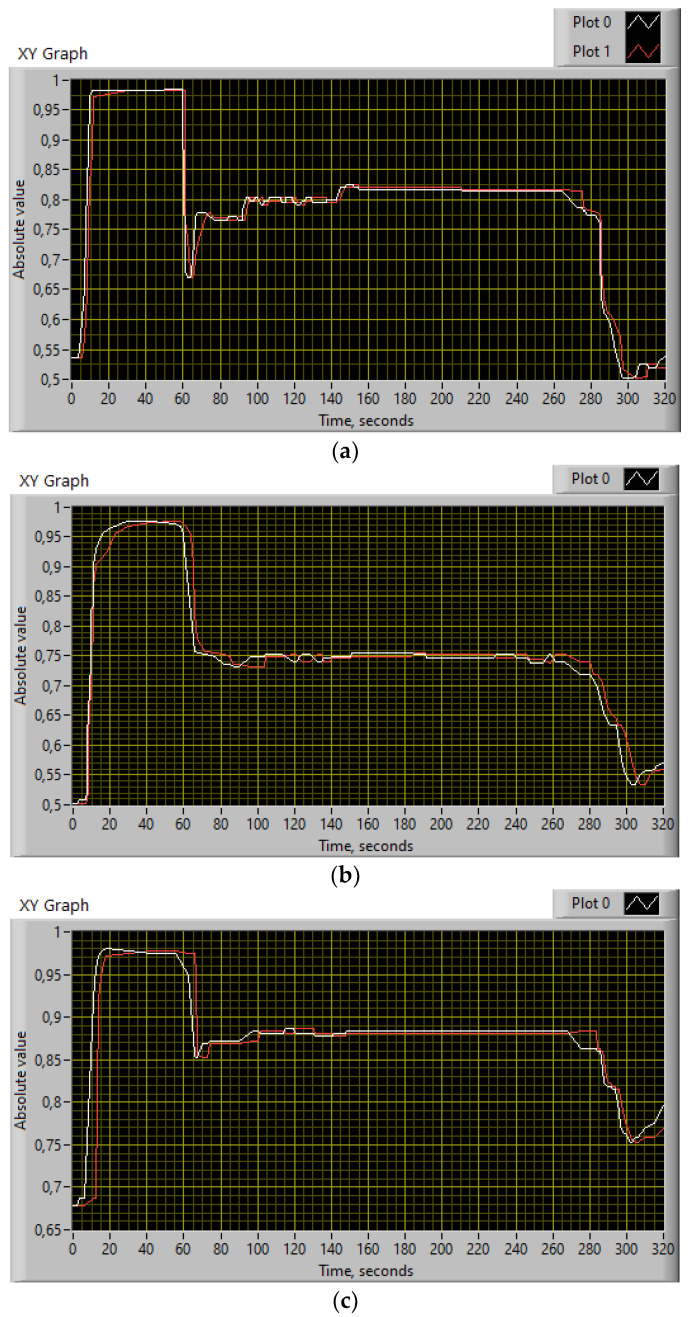
Diagram of the helicopter turboshaft engines parameters (using the TV3-117 engine as an example) after the low-frequency filtering procedure with an eighth-order Butterworth filter: (**a**): gas-generator rotor r.p.m; (**b**) free turbine rotor speed; (**c**) gas temperature in front of the compressor turbine (author’s research).

Figure 9 shows the low-frequency filtering results with an eighth-order Butterworth filter, implemented in the LabView software environment, of the training samples of the helicopter TE thermogas-dynamic parameters (Table 2).

To determine the low-frequency digital filter parameters, it is advisable to determine the spectral composition of the experimental data, for example, using fast Fourier transform (FFT) [63,64]:

(64) $$F\left(\omega \right)=\int_{-\infty }^{\infty }f\left(t\right)\cdot e^{-j\cdot \omega \cdot t}dt,$$

where it is necessary to take into account that when using Fourier transform to obtain informative spectral characteristics, it is necessary to subtract their average value from the experimental data:

(65) $$∆{\bar{y}}_{i}=y_{i}-\bar{y},$$

where *y_i_* is the experimental data at the *i*-th point and $\bar{y}$ is the average value according to the experimental data.

**Figure 10:**
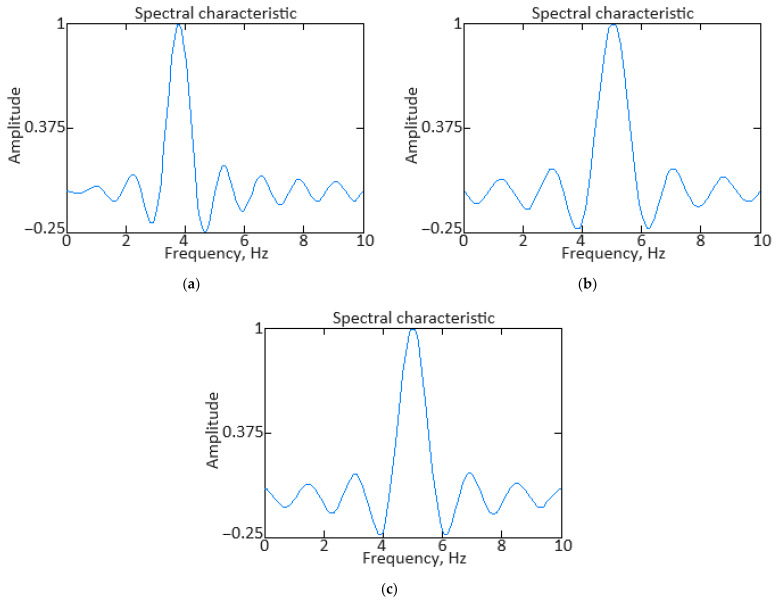
Diagrams of the spectral characteristics of the helicopter turboshaft engine parameters (using the TV3-117 engine as an example): (**a**) gas-generator rotor r.p.m., (**b**) free turbine rotor speed, (**c**) gas temperature in front of the compressor turbine (author’s research).

The values obtained using Equation (65) were used to obtain the spectral characteristics using fast Fourier transform. The spectral characteristics of the helicopter TE parameters *n_TC_*, *n_FT_*, and $T_{G}^{*}$ are shown in Figure 10.

**Figure 11:**
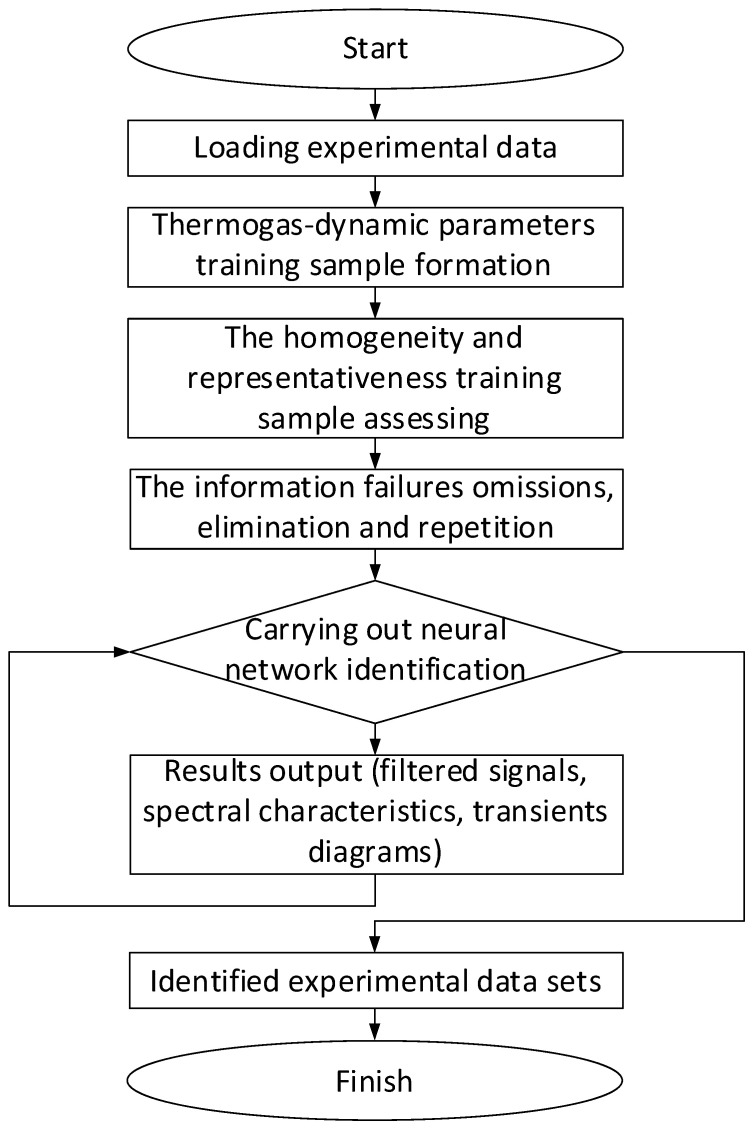
Scheme of the training sample loading of the helicopter turboshaft engine’s thermogas-dynamic parameters and the processing of experimental data by the proposed algorithm (author’s research).

The preliminary processing results of the training sample of the helicopter TE thermogas-dynamic parameters (Table 2) made it possible to synthesize the universal algorithm presented in Figure 11.

### 3.2. Results of the Helicopter Turboshaft Engine Nonlinear Dynamic Model at the Starting and Acceleration Modes in Neural Network Identification Experimental Research

To carry out neural network identification, we used the developed structure of the nonlinear dynamic model at the starting mode and experimental data on the thermogas-dynamic parameters *n_TC_*, *n_FT_*, and $T_{G}^{*}$ (Figure 7), obtained in the TV3-117 TE flight operation mode. According to the developed method for identifying a nonlinear dynamic model at starting mode, the experimental data were subjected to preliminary processing and a filtering procedure. Next, transient processes were approximated using a modified Elman neural network with dynamic stack memory (Figure 6).

Following on from the developed theoretical principles, the helicopter TE nonlinear dynamic model at the starting mode is a differential equations system (2). The mode parameter was formed as a numerical array corresponding to the time counts from the combustion chamber ignition until the idle gas mode was reached.

To determine the nonlinear dynamic model coefficients, the search starting point was determined. For this aim, the algebraic equations generated by overdetermined systems were solved by the least squares method. As a result, the following coefficients for the vector-matrix equations were obtained:

(66) $$X_{n_{TC}}=\left(-0.242;\;0.089;\;0.063;0.009\right);\;X_{n_{FT}}=\left(-0.137;-0.125;\;0.018;0.024\right);\;X_{T_{G}^{*}}=\left(-0.0061;\;0.0082;-0.0321;0.177\right);$$

**Figure 12:**
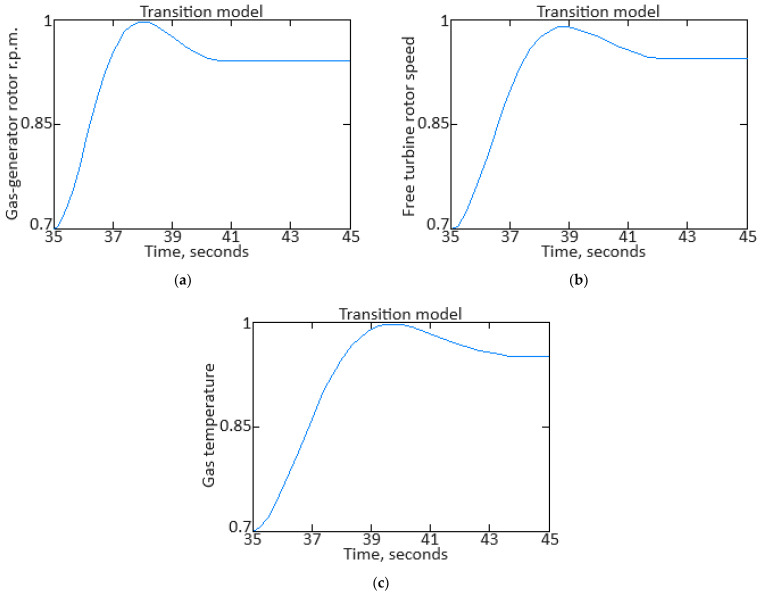
Diagrams of the transient processes of the helicopter turboshaft engine parameters at starting mode (using the TV3-117 engine as an example): (**a**) gas-generator rotor r.p.m., (**b**) free turbine rotor speed, (**c**) gas temperature in front of the compressor turbine (author’s research).

**Figure 13:**
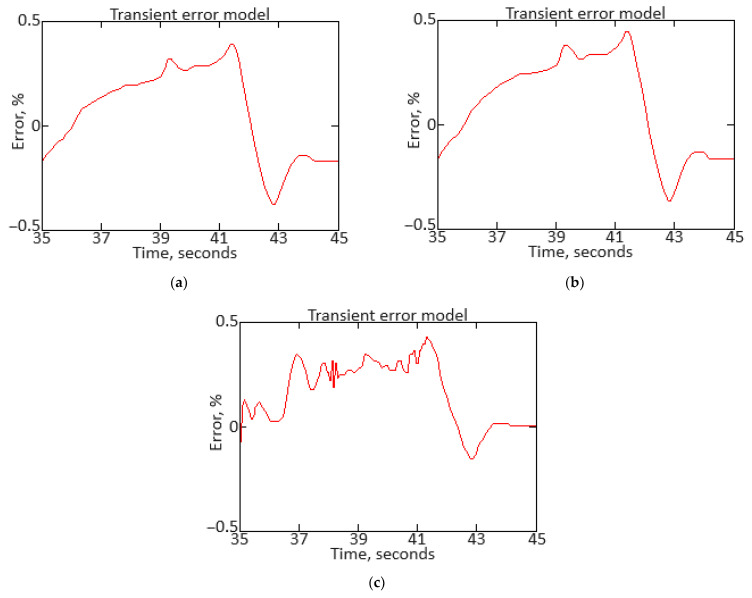
Diagrams of the difference between the simulated and experimental processes of the helicopter turboshaft engine at starting mode (using the TV3-117 engine as an example): (**a**) gas-generator rotor r.p.m., (**b**) free turbine rotor speed, (**c**) gas temperature in front of the compressor turbine (author’s research).

Next, with the given structure of the mathematical model, the coefficient values of the equations are calculated at each integration step, each value optimal for the mode parameter, at which the helicopter TE’s calculated output parameter is closest to its experimental value. As a result, when solving optimization tasks with a local optimality criterion, the equations’ coefficients were found for the mode parameter of each value. Based on the found coefficients, the functional dependencies on the mode parameter were determined. Next, the helicopter TE resulting dynamic model was checked, where the mathematical model is presented as (2). The resulting diagrams of the transient processes of parameters *n_TC_*(*t*), *n_FT_*(*t*), $T_{G}^{*}$(*t*), simulated using the helicopter TE (using the TV3-117 TE as an example), identified a nonlinear dynamic model at the starting mode, which are presented in Figure 12. Figure 13 shows the diagrams of the difference between the simulated and experimental processes.

The identifying results of the helicopter TE nonlinear dynamic model parameters at starting mode according to the developed method show that the identification error was less than 0.5%. In this case, the coefficients are presented in the form of the functional dependences on the mode parameter, which represent the time from combustion chamber ignition to reaching idle mode.

Similar studies were carried out on the helicopter TE (using the TV3-117 engine as an example) throttle response mode. In this case, the helicopter TE nonlinear dynamic model in the acceleration mode is a differential Equation (16) system. The mode parameter formed as a numeric array from the range (1–10), where 1 corresponds to idle mode and 10 to emergency mode. To determine the nonlinear dynamic model coefficients, the starting point was taken corresponding to the linear dynamic model coefficient values.

**Figure 14:**
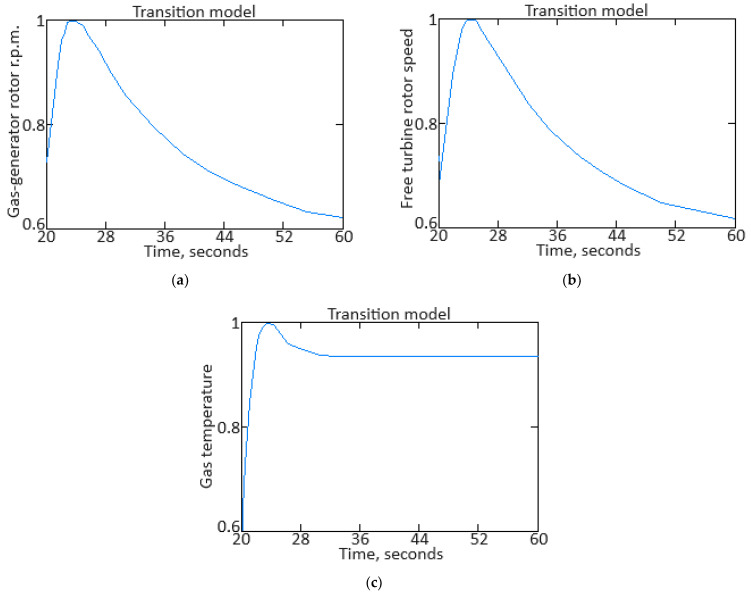
Diagrams of the transient processes of the helicopter turboshaft engine parameters at acceleration mode (using the TV3-117 engine as an example): (**a**) gas-generator rotor r.p.m., (**b**) free turbine rotor speed, (**c**) gas temperature in front of the compressor turbine (author’s research).

Next, with the given structure of the mathematical model, at each integration step, the equations’ coefficient values were calculated at which the helicopter TE calculated output parameter was closest to their experimental values. As a result, when solving optimization tasks with a local optimality criterion, the equations’ coefficients were determined. Based on the model calculated coefficients, their functional dependences on the mode parameter were determined. According to the methodology, the helicopter TE resulting dynamic model is then checked, where the mathematical model is presented in the form of Equation (16). The diagrams of the transient processes calculated using the model and experimental processes are presented in Figure 14.

**Figure 15:**
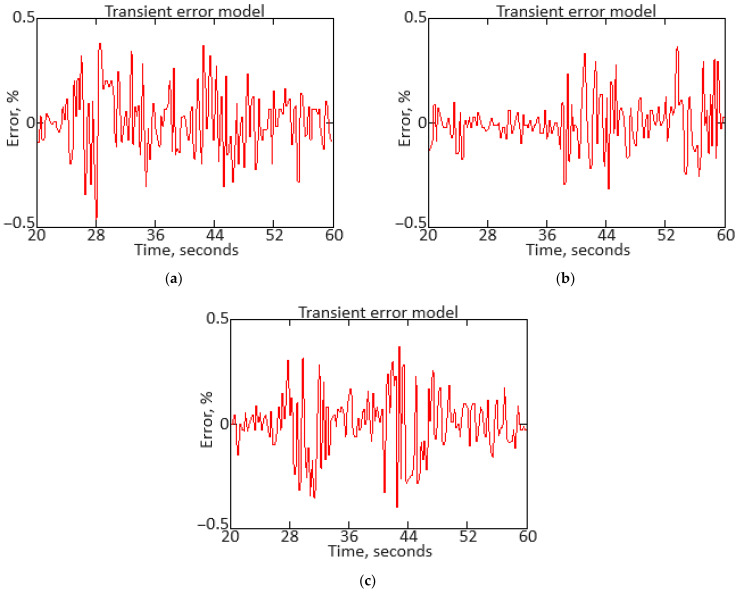
Diagrams of the difference between the simulated and experimental processes of the helicopter turboshaft engine at acceleration mode (using the TV3-117 engine as an example): (**a**) gas-generator rotor r.p.m., (**b**) free turbine rotor speed, (**c**) gas temperature in front of the compressor turbine (author’s research).

The diagrams of the difference between the simulated and experimental processes are presented in Figure 15. The resulting diagrams of the difference in the transient processes of parameters *n_TC_*(*t*), *n_FT_*(*t*), and $T_{G}^{*}$(*t*), obtained by calculation using the helicopter TE at the acceleration mode, identified the nonlinear dynamic model, and as presented in Figure 15, show that the identification error was less than 0.5%. In this case, the coefficients are presented in the form of functional dependencies on the mode parameter.

### 3.3. The Input Data Analysis and Pre-Processing Results

**Figure 16:**
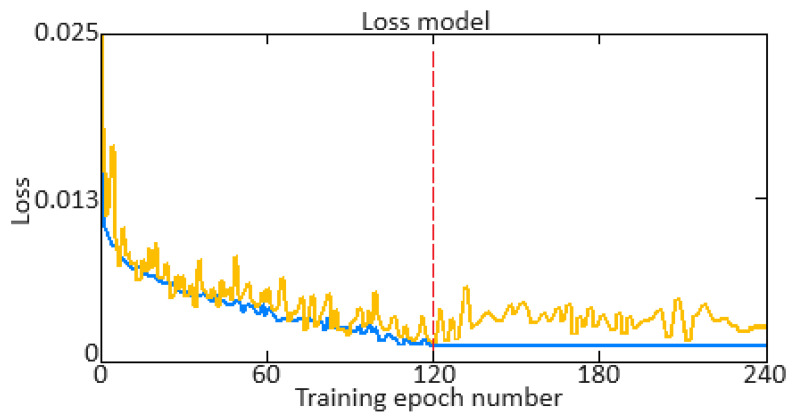
Diagram of the influence of epoch number passed the mean square error (author’s research).

**Figure 17:**
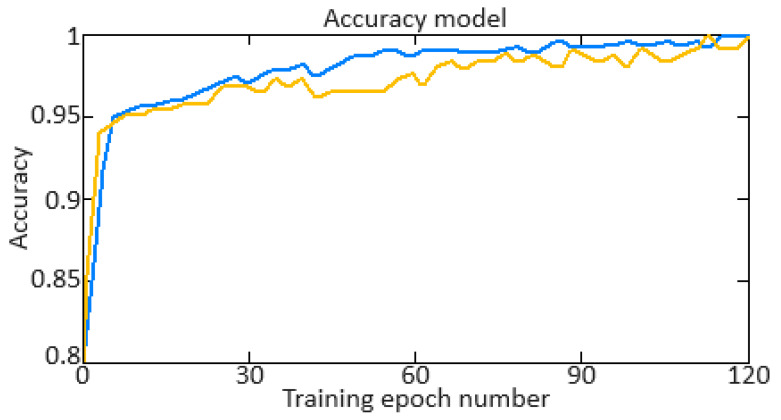
Diagram of accuracy metric (author’s research).

**Figure 18:**
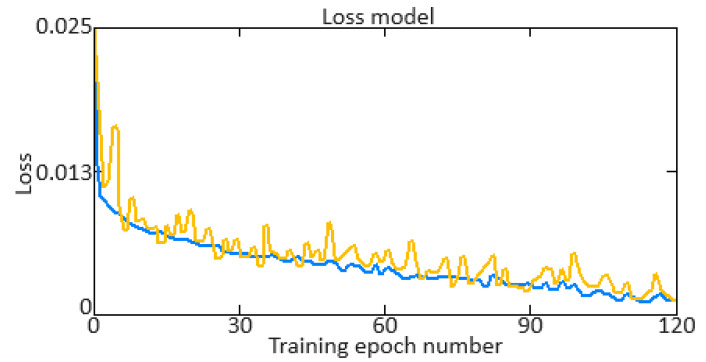
Diagram of loss function (author’s research).

**Figure 19:**
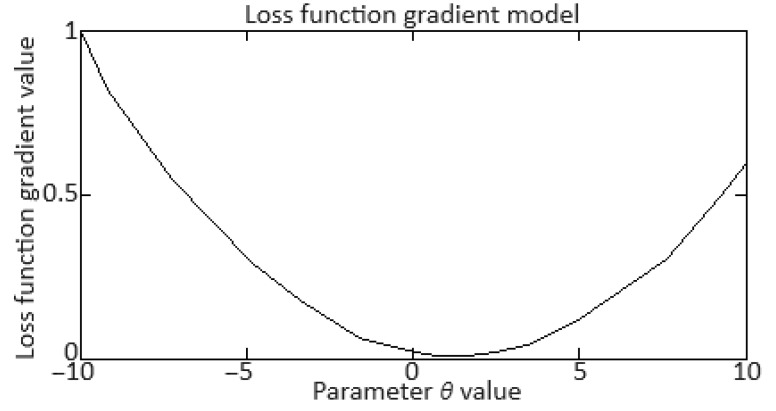
Diagram of changes in loss function gradients (author’s research).

The efficiency of the proposed Elman neural network with dynamic stack memory was assessed by determining the optimal number of training epochs according to Equation (41) (Figure 16), the accuracy metric according to Equation (42) (Figure 17), the loss function according to Equation (43) (Figure 18), and the loss function gradient diagram according to Equation (44) (Figure 19).

In Figure 16, the red dotted line indicates the model overfitting boundary, delineating the threshold beyond which the model begins to capture noise in the training data rather than the underlying data distribution. This boundary is critical for identifying the point at which the model’s complexity surpasses the optimal level, leading to a decline in generalization performance on unseen data. The careful monitoring of overfitting and mitigation at this boundary are essential for maintaining model robustness and predictive accuracy in real-world applications.

The slight increase in training error after the occurrence of *i*-th epoch due to model overtraining was thoroughly examined in [89,90,91]. These studies proposed several approaches to mitigate this issue, such as hyperparameter tuning and regularization techniques, aimed at stabilizing the training process and enhancing the model’s generalization capability. Notably, this temporary rise in training error is manageable with the right strategies, enabling the model to continue training and achieve optimal performance [92,93].

The results obtained indicate the following:

1. To achieve the minimum value *MSE_min_* = 0.00124, 120 training epochs were sufficient. At 120 epochs, the neural network training converged.
2. The neural network model had high accuracy (99.88%), which was confirmed by the resulting accuracy metric on both the training and test datasets.
3. The loss metric did not exceed 2.5%, while there was a significant decrease in the loss function from 2.5 to 0.12%. This indicates that the model not only accurately identified the helicopter TE nonlinear dynamic model parameters, but also demonstrated a high stability and reliability during the training process.
4. According to the diagram of the loss function gradients, the gradients decreased over time, indicating that the model approached the loss function minimum. The diagram, which had a U-shape [92,93] with *θ* varying from –10 to +10 and the gradient from 0 to 1, demonstrated the minimum gradient value (*θ* = 0.65) point, which indicates the model’s optimal parameter setting and high accuracy at this point. The resulting diagram with the minimum gradient value (*θ* = 0.65) indicates the stable training of the neural network as the gradient approaches zero, ensuring the model parameters’ accuracy and optimal adjustment.

Based on [34,73], the Elman network input vector represents the values of the variable parameters *n_TC_*(*t*), *n_FT_*(*t*), and $T_{G}^{*}$(*t*) as well as the signals at the output of the hidden layer neurons, delayed by the discrete time one clock cycle.

As in [34,73], we denoted the hidden layer neurons’ state vector as *V*, and the network output vector as *Y*. The Elman network input vector at time *k* implements the mapping as follows:

(67) $$U_{1}(k)=\{n_{TC}(k),V_{1}(k-1),V_{2}(k-1)\},\;U_{2}(k)=\{n_{FT}(k),V_{1}(k-1),V_{2}(k-1)\},\;U_{3}(k)=\{T_{G}^{*}(k),V_{1}(k-1),V_{2}(k-1)\}.$$

**Figure 20:**
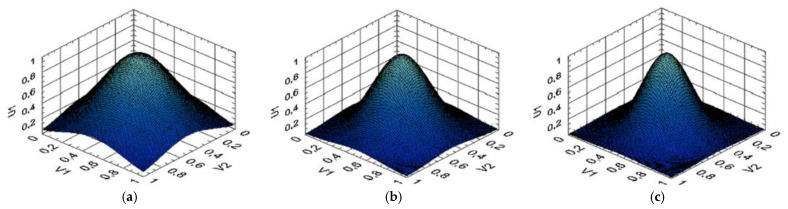
Diagrams of the distribution surfaces *U*_1_(*k*) (**a**), *U*_2_(*k*) (**b**), and *U*_3_(*k*) (**c**) (author’s research).

Figure 20 shows the distribution surfaces *U*_1_(*k*), *U*_2_(*k*), and *U*_3_(*k*) according to the *n_TC_*(*t*), *n_FT_*(*t*), and $T_{G}^{*}$(*t*) values given in the training set (Table 2). Figure 20 shows that the distribution surfaces *U*_1_(*k*), *U*_2_(*k*), *U*_3_(*k*) were flat without random bursts, which represents the network stability and predictability behavior during training and operation. The absence of sharp fluctuations in the distribution surfaces indicates a network parameter and its ability for high-quality selection to effectively process input data without significant anomalies or errors.

**Figure 21:**
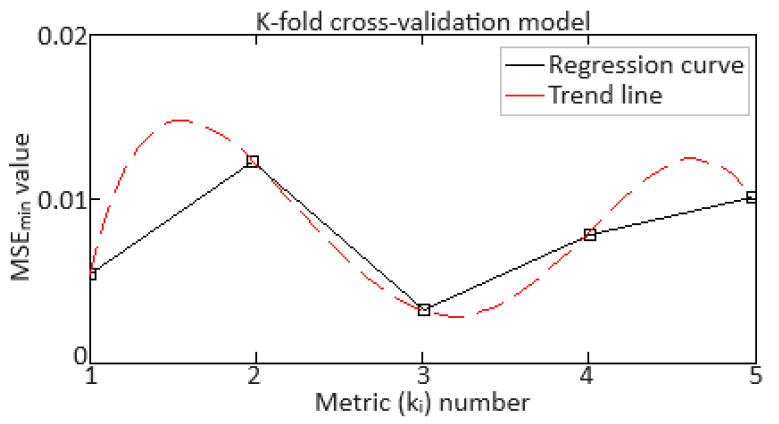
The resulting k-fold cross-validation diagrams (author’s research).

At the final stage of assessing the computational experiment’s effectiveness, a cross-validation procedure (k-fold cross-validation) was carried out. To implement this, *k* = 5 equal parts (folds) of 51 elements each were randomly selected from the training sample. The following metric values were obtained: *Metric*(*k*_1_) = 0.0012, *Metric*(*k*_2_) = 0.0015, *Metric*(*k*_3_) = 0.0011, *Metric*(*k*_4_) = 0.0013, *Metric*(*k*_5_) = 0.0014 (Figure 21, where the “black line” shows distribution of the corresponding *Metric*(*k_i_*) metric values, the “red curve” is the regression model). Then, the value of the *Average Metric* = 0.0013, which differed from the *MSE_min_* = 0.00124 by 4.65%. This means that the average value of the metric (mean square error) after cross-validation is close to the *MSE_min_* obtained on the folds (in this case, on *k*_1_). The 4.65% difference indicates how much the average metric value differs from the best individual result obtained in the cross-validation iterations.

The results also indicate that the model was quite stable in its predictions on different parts of the data (folds), since the difference between the *Average Metric* and *MSE_min_* was small. However, it is important to consider that the *Average Metric* provides more general information about its performance on the entire training set, while *MSE_min_* reflects the best result achieved on one part of the data.

As can be seen from Figure 21, the regression model has the form:

(68) $$y=-8\;\cdot \;10^{-5}\;\cdot \;n_{TC}+0.0011\;\cdot \;n_{FT}-0.0046\;\cdot \;T_{G}^{*}+0.0079\;\cdot \;G_{T}\;-\;0.0031.$$

The determination coefficient was 0.997, which means that the regression model fit the data in cross-validation almost perfectly.

Thus, the resulting regression model was configured in such a way that it almost perfectly identified the dependent variables (helicopter TE thermogas-dynamic parameters *n_TC_*(*t*), *n_FT_*(*t*), and $T_{G}^{*}$(*t*)) based on data from the cross-validation on each fold.

## 4. Discussion

Table 3 shows the comparative analysis results of the helicopter TE dynamic model identification using the proposed modified Elman neural network with dynamic stack memory, the traditional Elman neural network developed in [34,73], and the nonlinear parameter approximation using classical methods (cubic spline interpolation). To carry out a comparative analysis, metrics (48)–(57) were used.

The proposed modified Elman neural network demonstrated significant quantitative improvements across various metrics compared to both the traditional Elman neural network and cubic spline interpolation. The *R*^2^ value of 0.997 and $R_{adj}^{2}$ of 0.992 highlight a superior fit and explanatory power, surpassing the traditional Elman network (0.932 and 0.927, respectively) and cubic spline interpolation (0.717 and 0.713, respectively). The *MAE* of 0.035 for the proposed model indicated a much lower average error compared to 0.101 for the traditional Elman network and 0.213 for cubic spline interpolation. The correlation coefficient *r* of 0.983 further confirmed the high predictive accuracy of the proposed model, significantly higher than 0.901 and 0.702 for the traditional and cubic spline methods, respectively. The *relative error* (*RE*) of 0.036 was markedly better than 0.104 and 0.219, respectively. Additionally, *Foote’s metric* (*FM*) was 0.965, indicating better segmentation quality over 0.889 and 0.637, respectively. The proposed model also exceled in classification metrics, with an *Accuracy* of 0.998, *Precision* of 0.991, *Recall* of 1.0, and *F1-score* of 0.995, outperforming the traditional network and cubic spline interpolation across all metrics, thus demonstrating its superior overall performance.

For the four classification classes (True Positives, True Negatives, False Positives, False Negatives), a confusion matrix was developed (Table 4). The confusion matrix for each cell indicates the number of times the actual class (rows) was classified as the predicted class (columns) for each method.

The confusion matrix revealed that the proposed modified Elman neural network with dynamic stack memory was the most accurate method, successfully classifying the majority of instances in all classes with minimal errors. In comparison, the traditional Elman neural network developed in [34,73] performed well, but exhibited slightly more errors, particularly in differentiating between the True Negative and False Positive classes. The cubic spline interpolation showed moderate accuracy but had noticeable misclassifications across all classes, especially in identifying instances of False Positives. Overall, the proposed modified Elman neural network with dynamic stack memory outperformed the other methods, followed by the traditional Elman neural network, and finally, the cubic spline interpolation in terms of classification quality.

To perform ROC analysis for the three methods (proposed modified Elman neural network with dynamic stack memory, traditional Elman neural network, and cubic spline interpolation), the True Positive and False Positive rates were calculated for each class and method, then the corresponding ROC curves were generated. This involved creating a binary classification for each class (this class versus all others). Table 5 shows the results of the ROC analysis.

**Figure 22:**
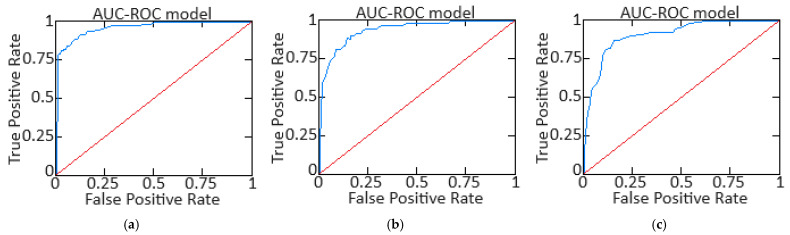
The AUC-ROC curves. (**a**) Proposed modified Elman neural network with dynamic stack memory; (**b**) traditional Elman neural network developed in [34,66]; (**c**) cubic spline interpolation (author’s research).

Thus, the proposed modified Elman neural network with dynamic stack memory provides high accuracy with a low level of False Positive results; the traditional Elman neural network also provided high accuracy, but was 1.16 times lower than that of the proposed modified Elman neural network with dynamic stack memory. Using cubic spline interpolation provided moderate accuracy, with noticeable errors. Using cubic spline interpolation also gave the lowest accuracy, with the highest number of False Positives. Figure 22 clearly demonstrates the difference in the areas under the AUC curve.

At the next stage of comparative analysis, the first and second type errors were assessed in the helicopter TE dynamic model identifying task. Table 6 shows the calculated results of the first and second type errors for the modified Elman neural network with dynamic stack memory, the traditional Elman neural network, and cubic spline interpolation.

As can be seen from Table 6, the developed modified Elman neural network with dynamic stack memory use made it possible to reduce the first and second kind errors by 1.5 times compared with the traditional Elman neural network and by 3.25 times compared with the cubic spline interpolation.

In the course of the experimental research, a comparative analysis of the neural network (the developed modified Elman neural network with dynamic stack memory and the traditional Elman neural network) and classical (the cubic spline interpolation) methods’ absolute error determination was carried out under white noise conditions (with zero mathematical expectation *M* = 0 and values *σ_T_* = 0.01, 0.03, 0.05). The results obtained are shown in Table 7.

The results obtained indicate the advantage of neural network methods (using the developed modified Elman neural network with dynamic stack memory) over classical ones (the cubic spline interpolation). In some cases, the dynamic identification errors using the classical method were almost twice as large as the corresponding calculations using a modified Elman neural network with dynamic stack memory, which indicates the neural networks’ high stability to external disturbances. Therefore, based on the results of the analytical and experimental research, the following conclusions can be drawn:

1. The use of the developed modified Elman neural network with dynamic stack memory allowed us to solve the helicopter TE dynamic model identifying task with an absolute error of no more than 0.578%.
2. The accuracy of the dynamic identification of the helicopter TE parameters (using the TV3-117 TE as an example) based on a modified Elman neural network with dynamic stack memory was on average 1.70 times higher than the traditional Elman neural network developed in [34,66], and 6.67 times higher than the cubic spline interpolation.

The reliability of the developed modified Elman neural network with dynamic stack memory was evaluated by examining its performance across various datasets and conditions. Robustness is assessed by analyzing the changes in reconstruction accuracy when the input data or model parameters are varied [77]. This research used a conventional method for evaluating neural network stability, the standard deviation accuracy calculated in the helicopter TE parameters’ (using the TV3-117 TE as an example) dynamic identification task across different subsamples or datasets. A smaller standard deviation indicates greater resilience to data or condition changes. The standard deviation accuracy of the helicopter TE parameters’ (using the TV3-117 TE as an example) dynamic identification task is given by the expression:

(69) $$\sigma =\sqrt{\frac{\sum_{i=1}^{N}{\left({Acc}_{i}-\bar{Acc}\right)}^{2}}{n}},$$

where *Acc_i_* is the neural network accuracy on the *i*-th dataset or with the variation in *i*-th parameters, $\bar{Acc}$ is the average accuracy value all observations, and *n* is the number of subsamples.

In this work, for each neural network, the accuracy was determined on *n* = 8 different subsamples of 32 values in each training sample with a total size of 256 values. It was assumed that *Acc*_1*i*_ and *Acc*_2*i*_ were the neural network accuracy values for the modified Elman neural network with dynamic stack memory and the traditional Elman neural network on the *i*-th subsample, respectively (Table 8). The average accuracy for each neural network was $\bar{{Acc}_{1}}$ = 0.998 and $\bar{{Acc}_{2}}$ = 0.954, and the average deviation was 0.00738 and 0.01991, respectively.

A decrease in the average deviation by 2.7 times indicates that the proposed modified Elman neural network with dynamic stack memory was almost two times more robust compared to the traditional Elman neural network, which indicates its ability to maintain high accuracy across different datasets and conditions.

To assess the performance of the modified Elman neural network with dynamic stack memory in terms of efficiency, this research employed the *Efficiency* metric, which accounts for both prediction accuracy and the amount of resources expended during training and/or inference. This metric can be expressed as the relationship between accuracy and resource consumption (such as computational power or time resources). Suppose *Acc*_1_ and *Acc*_2_ represent accuracies of the two neural networks being compared (the modified Elman neural network with dynamic stack memory and the traditional Elman neural network), and *Res*_1_ and *Res*_2_ denote the resources utilized (in this research, the training time parameter was used, as both networks were tested with an identical memory capacity of 32 GB DDR-4). The resource efficiency (Table 9) usage can then be determined by the following expression:

(70) $${Efficiency}_{1}=\frac{{Acc}_{1}}{{Res}_{1}},{\;\;\;Efficiency}_{2}=\frac{{Acc}_{2}}{{Res}_{2}}.$$

As demonstrated by the computational experiment results focused on analyzing the efficiency of resource allocation, implementing dynamic stack memory increased resource utilization by 2.33%. Despite the seemingly modest improvement of only 2.33%, this enhancement plays a critical role in systems handling large datasets, where even minor optimizations can significantly reduce the overall task execution time and improve system stability. This method can be effectively scaled for larger systems due to the inherent adaptability of the modified Elman neural network with dynamic stack memory, which allows for the efficient handling of increased data complexity and size. Moreover, the scalability is supported by the network’s capacity to manage higher computational loads without significant loss in accuracy, making it suitable for applications in more complex and expansive systems.

Thus, we proposed a method of determining the nonlinear dynamic model for a helicopter TE, which, due to a modified Elman neural network with a dynamic stack memory, enabled us to identify the helicopter TE’s nonlinear dynamic model in both starting and acceleration modes with an error standard deviation of 2.7 less compared to the analog [34]. This made it possible to increase the reliability of the identification of the helicopter TE’s nonlinear dynamic model by two, which indicates its ability to maintain high accuracy with different datasets and conditions.

The proposed helicopter TE dynamic model, based on a modified Elman neural network with dynamic stack memory, holds significant potential for practical applications in real-world helicopter operations. Its ability to accurately capture the complex dynamics of transitional modes such as starting and acceleration suggests that the model can be extended to other critical engine operating states, thus improving real-time engine performance monitoring and fault detection. This approach can enhance flight safety and operational efficiency by providing more reliable diagnostic capabilities and early warning systems for detecting anomalies during various flight regimes. Furthermore, the underlying architecture of the model, particularly the use of dynamic stack memory, offers adaptability, which may be applicable to other aircraft systems beyond helicopter engines. For instance, this method could potentially be employed in other propulsion systems, such as jet engines or in auxiliary power units (APUs), where dynamic behavior and the need for real-time response to changing conditions are critical. In commercial aviation, where the engines’ reliability and safety are paramount, this model could contribute to more precise predictive maintenance systems, reducing unscheduled downtimes and operational costs. The proposed neural network architecture’s flexibility suggests that, with appropriate modifications and further validation, it could be applied across a range of aircraft systems, leading to broader adoption within both the military and civil aviation sectors.

While the helicopter TE proposed dynamic model, developed using a modified Elman neural network with dynamic stack memory, demonstrated high accuracy in capturing the nonlinear dynamics of transitional modes, certain limitations should be acknowledged. One key limitation arises from the nonlinear model’s inherent complexity, which, while essential for accurately representing the intricate behavior of helicopter TEs, also increases the computational requirements and may limit real-time applicability in some high-demand environments. Additionally, the model’s current validation was performed using parameters and a data specific set related to the helicopter TE starting and acceleration phases. This introduces constraints on the model’s generalization to other operating modes or engine types without further adaptation and testing. Therefore, while the model exhibited strong performance within the scope of the evaluated scenarios, its broader validity may be limited if applied to significantly different operational contexts or aircraft systems without reconfiguring the neural network’s structure or retraining with a more diverse dataset.

Further research will focus on substantiating the effectiveness of the developed helicopter TE dynamic model, based on the modified Elman neural network with dynamic stack memory, through extended experiments aimed at testing its applicability across a broader range of transitional modes. These experiments will not only encompass starting and acceleration modes, on which the model has already been successfully tested, but also other key operational modes such as stabilization at various power levels, transitions between different thrust regimes, and engine behavior during short-term failures and external disturbances. Expanding the scope of tested states will enable an evaluation of the model’s ability to control dynamic changes in engine parameters and respond promptly to non-standard or emergency situations. Special attention will be given to testing the model under changing external factors such as temperature and pressure fluctuations as well as in the presence of random measurement errors, allowing for an assessment of its robustness and accuracy in real operational conditions. It is anticipated that the results of these experiments will confirm the proposed model’s versatility and reliability, marking an important step toward its integration into on-board intelligent systems for the real-time control and monitoring of helicopter TEs.

## 5. Conclusions

The authors developed a dynamic model for starting and acceleration modes by employing on-board parameters recorded by sensors (gas-generator rotor r.p.m., free turbine rotor speed, gas temperature in front of the compressor turbine, fuel consumption). The received results are summarized are follows:

1. Since helicopter turboshaft engines operate at steady-state modes about 85% of the time and only about 15% at unsteady and transient modes, to expand control, an urgent scientific and practical task is to develop dynamic multi-mode models for helicopter turboshaft engines that take into account the engine’s behavior at unsteady and transient modes.
2. The helicopter turboshaft engine dynamic model at starting mode and engine acceleration was further developed by taking into account the engine parameters recorded on-board the helicopter (gas-generator rotor r.p.m., free turbine rotor speed, gas temperature in front of the compressor turbine, fuel consumption), which made it possible to identify these parameters’ dynamics in unsteady and transient modes (starting mode and engine acceleration) with a 99.88% accuracy.
3. The Elman recurrent neural network was improved by adding dynamic stack memory, which made it possible, by increasing the neural network robustness, to increase the performance of solving the helicopter turboshaft engine dynamic model’s neural network identification task by 2.7 times compared to the traditional Elman neural network.
4. A theorem was proposed and proven, that in the proposed mathematical model of dynamic stack memory by adding (Push) and deleting (Pop) operations, the total execution time for *N* elements does not exceed a certain value *O*(*N*). This means that when *N* operations are performed, no matter how many times the stack is expanded or contracted, the total time taken to complete all Push and Pop operations will be proportional to *N*. Thus, on average, each operation completes in constant *O*(1) time, which provides efficient memory management and high performance, even with heavy stack usage.
5. The training algorithm of the Elman recurrent neural network with dynamic stack memory was improved, which, due to the initial preparation of the input data taking into account time delays and preliminary filtering using an *n*-th order Butterworth filter, allowed for 120 training epochs to reduce the maximum value of the loss function from 2.5 to 0.12% as well as ensured solving of the helicopter turboshaft engine dynamic model’s neural network identification task up to 99.88%.
6. It was established that, according to the developed loss function gradient diagram, that the gradients decreased over time, which indicates that the model approached the loss function minimum. The diagram, which had a U-shape with a change in *θ* from –10 to +10 and a gradient from 0 to 1, demonstrated the minimum value point of the gradient (*θ* = 0.65), which indicates the optimal setting of model parameters and high accuracy, ensuring that the recurrent neural Elman networks with dynamic stack memory have stable training as the gradient approaches zero.

Thus, we proposed a method of determining the nonlinear dynamic model for a helicopter TE, which, due to a modified Elman neural network with a dynamic stack memory, enabled us to identify the helicopter TE nonlinear dynamic model in both starting and acceleration modes with significantly lower error standard deviation in comparison with the analog [34].

One of our prospective future research directions include the integration of the developed helicopter TE neural network dynamic model into the helicopter TE on-board neural network expert system for monitoring and operation control [93] in the form of a neural network module. This integration will enable real-time monitoring and control of helicopter TE performance, enhancing system adaptability and decision-making accuracy. By embedding the neural network dynamic model into the on-board expert system, operators can achieve improved diagnostic capabilities, allowing for the proactive maintenance and optimization of engine operations, ultimately leading to increased reliability and safety in flight.

## Figures and Tables

**Table 1:** The error matrix.

Predicted Classes/True Classes	Class 1: Stationary Mode	Class 2: Transient Mode	Class 3: Unstable Mode
**Class 1: Stationary mode**	*TP*	*FP*	*FP*
**Class 2: Transient mode**	*FN*	*TP*	*FP*
**Class 3: Unstable mode**	*FN*	*FN*	*TP*

**Table 2:** The training sample fragment (author’s research).

Value	1	…	45	…	93	…	156	…	204	…	232	…	256
***n**_**TC**_*	0.686	…	0.973	…	0.902	…	0.905	…	0.906	…	0.738	…	0.823
***n**_**FT**_*	0.532	…	0.988	…	0.744	…	0.758	…	0.759	…	0.487	…	0.505
$T_{G}^{*}$	0.488	…	0.984	…	0.711	…	0.719	…	07.20	…	0.502	…	0.521

**Table 3:** The results of the comparative analysis (author’s research).

Metric	Proposed Modified Elman Neural Network	Traditional Elman Neural Network	Cubic Spline Interpolation
*R* ^2^	0.997	0.932	0.717
$R_{adj}^{2}$	0.992	0.927	0.713
*MAE*	0.035	0.101	0.213
*r*	0.983	0.901	0.702
*RE*	0.036	0.104	0.219
*FM*	0.965	0.889	0.637
*Accuracy*	0.998	0.954	0.837
*Precision*	0.991	0.942	0.826
*Recall*	1.0	1.0	0.763
*F1-score*	0.995	0.970	0.793

**Table 4:** The confusion matrix (author’s research).

Actual/Predicted	Proposed Modified Elman Neural Network	Traditional Elman Neural Network	Cubic Spline Interpolation
True Positives	94	3	3
True Negatives	3	88	5
False Positives	1	6	89
False Negatives	0	3	9

**Table 5:** Results of the ROC analysis (author’s research).

Actual/Predicted	Proposed Modified Elman Neural Network	Traditional Elman Neural Network	Cubic Spline Interpolation
True Positives	94	87	3
True Negatives	4	10	7
False Positives	272	260	280
False Negatives	28	41	108
True Positive Rate	0.832	0.785	0.036
False Positive Rate	0.012	0.023	0.068
AUC	0.892	0.811	0.647

**Table 6:** The results of the first and second type errors calculated from the helicopter TE dynamic model identification task (author’s research).

Error Type	Proposed Modified Elman Neural Network	Traditional Elman Neural Network	Cubic Spline Interpolation
Type I error,%	0.692	1.038	2.249
Type II error,%	0.401	0.602	1.303

**Table 7:** The comparative analysis of the absolute error calculation in identifying the helicopter turboshaft engine dynamic model parameters under white noise conditions (author’s research).

Method	Absolute Error,% (*σ_T_* = 0.01)			Absolute Error,% (*σ_T_* = 0.03)			Absolute Error,% (*σ_T_* = 0.05)		
	*n_TC_*	*n_FT_*	$T_{G}^{*}$	*n_TC_*	*n_FT_*	$T_{G}^{*}$	*n_TC_*	*n_FT_*	$T_{G}^{*}$
Proposed modified Elman neural network	0.385	0.382	0.383	0.501	0.497	0.498	0.578	0.573	0.575
Traditional Elman neural network	0.653	0.648	0.651	0.849	0.842	0.846	0.980	0.972	0.977
Cubic spline interpolation	2.565	2.542	2.557	3.335	3.305	3.324	3.848	3.813	3.836

**Table 8:** The accuracy indicators of the calculation results of the proposed modified Elman neural network with dynamic stack memory with a famous network in the helicopter TE parameters’ (using the TV3-117 TE as an example) dynamic identification task, when dividing a training sample of 256 elements into 8 subsamples of 32 elements each.

Number of Subsamples	1	2	3	4	5	6	7	8	$\bar{Acc}$
**Proposed modified Elman neural network with dynamic stack memory**	0.998	0.998	0.997	0.997	0.999	0.999	0.998	0.998	0.998
**The closest analog (the traditional Elman neural network)**	0.952	0.942	0.943	0.964	0.966	0.950	0.950	0.950	0.954

**Table 9:** The result of the resource efficiency calculations of the proposed modified Elman neural network with dynamic stack memory and the traditional Elman neural network.

Parameter	Proposed Modified Elman Neural Network with Dynamic Stack Memory	The Closest Analog (the Traditional Elman Neural Network)
Accuracy (*Acc*)	0.998	0.954
Training rate (*Res*)	2 min 17 s–137 s	2 min 14 s–134 s
Efficiency	0.00729	0.00712

## Data Availability

Data are contained within the article.

## Appendix A

Dynamic stack memory allows for the efficient management of data during algorithm execution, especially with regard to recursive and hierarchical processes. The proposed model takes into account such aspects and offers a unique approach to stack memory management.

The basic parameters of the dynamic stack memory mathematical model are as follows: *S*(*t*) is the stack at time *t*, *H*(*t*) is the pointer to the stack top at time *t*, and *D*(*t*) is the data written to the stack top at time *t*.

The basic operations of the dynamic stack memory mathematical model: Push(*x*, *t*) is adding element *x* to the stack top at time *t*, Pop(*t*) is removing an element from the stack top at time *t*, Peek(*t*) is the peek at the element at the stack top without removing it.

Operations are formalized as follows:

1. Operation Push(*x*, *t*):
  *S*(*t*) = *S*(*t* − 1) + *x*, *H*(*t*) = *H*(*t* − 1) + 1, *D*(*t*) = *x*. (A1)
2. Operation Pop(*t*):
  (A2) $$S\left(t\right)=\frac{S\left(t-1\right)}{D\left(t-1\right)},\;H\left(t\right)=H\left(t-1\right)-1,\;D\left(t\right)=S\left(H\left(t\right)\right).$$
3. Operation Peek(*t*):
  (A3) $$D\left(t\right)=S\left(H\left(t-1\right)\right).$$

Initialization of memory management is carried out as:

(A4) $$S\left(0\right)=\emptyset ,\;H\left(0\right)=0,\;D\left(0\right)=\emptyset .$$

During dynamic memory management, to ensure the efficient use of memory, the stack can be divided into segments, where each segment has its own maximum size *M*.

The stack automatic expansion and contraction is conducted as follows:

1. If *H*(*t*) > *M*, then a new segment is created.
2. If *H*(*t*) < $\frac{M}{2}$, and there is more than one segment, then the stack is compressed and the segments are merged.

Example:

The stack is assumed to contain data about the events and timestamps of the helicopter TE control system.

1. Time *t* = 1 s:
  Push: *S*(1) = {*t*_1_}, *H*(1) = 1, *D*(1) = *t*_1_.
2. Time *t* = 2 s:
  Push: *S*(2) = {*t*_1_, *t*_2_}, *H*(2) = 2, *D*(2) = *t*_2_.
3. Time *t* = 3 s:
  Push: *S*(3) = {*t*_1_}, *H*(3) = 1, *D*(3) = *t*_1_.

This model can be used for:

1. Data management in recursive algorithms.
2. Memory implementations in neural networks using stacks to store states.

Resource management in real-time systems such as helicopter TE control systems requires fast and efficient state management.

The developed dynamic stack memory mathematical model differs from the traditional model by introducing a segmented structure for memory management, which allows for the stack to automatically expand and contract, depending on its fullness. In the traditional model, the stack is implemented as a contiguous array or list, where the Push(*x*, *t*) and Pop(*t*) operations change the pointer to the stack top without considering segmentation. In contrast, the proposed model uses fixed segments with a maximum size *M*, automatically creating new segments when the limit is exceeded, and merging segments when the data are significantly reduced. This approach provides more efficient memory usage and improves performance in stack-intensive systems such as neural networks and real-time systems.

**Theorem 1.** 
*In the dynamic stack memory mathematical model (A1)–(A4), the total execution time for N elements in the Push and Pop operations does not exceed a certain value O(N).*

**Proof of Theorem 1.** At the initial stage, the segments are defined. For this, it is assumed that each stack segment has a maximum size *M*. When performing a Push operation:

1. If the current segment is not full (*H*(*t*) < *M*):
  Push(*x*, *t*): *S*(*t*) = *S*(*t* − 1) + *x*, *H*(*t*) = *H*(*t* − 1) + 1, *D*(*t*) = *x*, (A5)
  which requires *O*(1) time to complete.
2. If the current segment is full (*H*(*t*) = *M*), then a new segment is created:
  Push(*x*, *t*): *S*(*t*) = {*x*}, *H*(*t*) = 1, *D*(*t*) = *x*, (A6)
  which requires *O*(1) time to complete.

When performing a Pop operation:

1. If the current segment is not empty (*H*(*t*) > 0):
  (A7) $$\mathrm{Pop}(t):\;S(t)=\frac{S\left(t-1\right)}{D\left(t-1\right)},H(t)=H\left(t-1\right)-1,D(t)=S(H(t))$$
  which requires *O*(1) time to complete.
2. If the current segment is empty (*H*(*t*) = 0) and previous segments exist, then a merger with the previous segment is performed
  Pop(*t*): *S*(*t*) = {Previous segment}, *H*(*t*) = *M* − 1, *D*(*t*) = *S*(*H*(*t*)), (A8)
  which requires *O*(1) time to complete.

Then, to determine the operations’ total execution time, *k* is the number of segments created for *N* operations. Then:

(A9) $$k\leq \left[\frac{N}{M}\right].$$

Then, the time to create segments is determined as:

(A10) $$O\left(k\right)=O\left(\frac{N}{M}\right)=O\left(N\right).$$

Then, the total time is:

(A11) $$\left\{\begin{array}{c} \mathrm{Time}\;\mathrm{for}\;\mathrm{each}\;\mathrm{Push}\;\mathrm{and}\;\mathrm{Pop}\;\mathrm{operation}:O\left(1\right)\\ \mathrm{Total}\;\mathrm{time}\;\mathrm{for}\;N\;\mathrm{operations}:\;O\left(N\right) \end{array}\right.$$

Therefore, the total execution time of the *N* Push and Pop operations in the proposed dynamic stack memory model does not exceed a certain value *O*(*N*), which is what needed to be proven. □

## Appendix B

The mean square error (*MSE*) function gradient is a set of vectors showing the direction and magnitude of the change in error as a change function in model parameter *θ*. It is known that the *MSE* formula has the form:

(A12) $$MSE=\frac{1}{N}\cdot \sum_{i=1}^{N}{\left(y_{i}-{\hat{y}}_{i}\right)}^{2}.$$

To calculate the *MSE* gradient with respect to the parameter *θ*, at the first stage, the *MSE* partial derivative with respect to *θ* is determined:

(A13) $$\frac{\partial MSE}{\partial \theta }=\frac{\partial }{\partial \theta }\cdot \left(\frac{1}{N}\cdot \sum_{i=1}^{N}{\left(y_{i}-{\hat{y}}_{i}\right)}^{2}\right).$$

It is assumed that ${\hat{y}}_{i}$ depends on *θ*. Then,

(A14) $$\frac{\partial MSE}{\partial \theta }=\frac{1}{N}\cdot \sum_{i=1}^{N}{\frac{\partial }{\partial \theta }\cdot \left(y_{i}-{\hat{y}}_{i}\right)}^{2}=\frac{1}{N}\cdot \sum_{i=1}^{N}2\cdot \left(y_{i}-{\hat{y}}_{i}\right)\cdot \left(-\frac{\partial {\hat{y}}_{i}}{\partial \theta }\right)$$

or

(A15) $$\frac{\partial MSE}{\partial \theta }=\frac{1}{N}\cdot \sum_{i=1}^{N}{\frac{\partial }{\partial \theta }\cdot \left(y_{i}-{\hat{y}}_{i}\right)}^{2}=-\frac{2}{N}\cdot \sum_{i=1}^{N}\left(y_{i}-{\hat{y}}_{i}\right)\cdot \frac{\partial {\hat{y}}_{i}}{\partial \theta }.$$

The gradient calculation result shows how much the *MSE* changes with a small change in the parameter *θ*. Gradient vectors indicate the change direction and magnitude that must be made to the model parameter *θ* to minimize the error.

## References

[1] Balli O. (2023). Exergetic, sustainability and environmental assessments of a turboshaft engine used on helicopter. Energy.

[2] Zhang S., Ma A., Zhang T., Ge N., Huang X. (2024). A Performance Simulation Methodology for a Whole Turboshaft Engine Based on Throughflow Modelling. Energies.

[3] Ahmadian N., Khosravi A., Sarhadi P. (2017). Adaptive control of a jet turboshaft engine driving a variable pitch propeller using multiple models. Mech. Syst. Signal Process..

[4] Zheng X., Zeng H., Wang B., Wen M., Yang H., Sun Z. (2023). Numerical simulation method of surge experiments on gas turbine engines. Chin. J. Aeronaut..

[5] Chen M., Qu R., Fang W. (2022). Case-based reasoning system for fault diagnosis of aero-engines. Expert Syst. Appl..

[6] Kim S., Im J.H., Kim M., Kim J., Kim Y.I. (2023). Diagnostics using a physics-based engine model in aero gas turbine engine verification tests. Aerosp. Sci. Technol..

[7] Hosseinimaab S.M., Tousi A.M. (2022). Optimizing the performance of a single-shaft micro gas turbine engine by modifying its centrifugal compressor design. Energy Convers. Manag..

[8] Liang W., Guan W., Ding Y., Hang C., Zhou Y., Zou X., Yue S. (2024). Mechanical Properties and Fatigue Life Analysis of Motion Cables in Sensors under Cyclic Loading. Sensors.

[9] Ye Y., Wang Z., Zhang X. (2021). Cascade ensemble-RBF-based optimization algorithm for aero-engine transient control schedule design optimization. Aerosp. Sci. Technol..

[10] Chen Q., Sheng H., Liu T. (2023). Fuzzy logic-based adaptive tracking weight-tuned direct performance predictive control method of aero-engine. Aerosp. Sci. Technol..

[11] Zheng J., Chang J., Ma J., Yu D. (2021). Modeling and analysis of windmilling operation during mode transition of a turbine-based-combined cycle engine. Aerosp. Sci. Technol..

[12] Pang S., Li Q., Ni B. (2021). Improved nonlinear MPC for aircraft gas turbine engine based on semi-alternative optimization strategy. Aerosp. Sci. Technol..

[13] Diniz M.M., Gomes L.T., Bassanezi R.C. (2021). Optimization of fuzzy-valued functions using Zadeh’s extension principle. Fuzzy Sets Syst..

[14] Kupka J. (2016). On approximations of Zadeh’s extension principle. Fuzzy Sets Syst..

[15] Li Z., Nikolaidis T., Nalianda D. (2016). Recursive Least Squares for Online Dynamic Identification on Gas Turbine Engines. J. Guid. Control Dyn..

[16] Tsoutsanis E., Meskin N., Benammar M., Khorasani K. (2016). A dynamic prognosis scheme for flexible operation of gas turbines. Appl. Energy.

[17] Liu N., Zhang X., Guo J., Chen S. (2023). Aero-Engine Remaining Useful Life Prediction Based on Bi-Discrepancy Network. Sensors.

[18] Pu X., Liu S., Jiang H. Observable degree analysis of heavy-duty gas turbine based on SVD method. Proceedings of the 2011 Second International Conference on Mechanic Automation and Control Engineering.

[19] Aygun H., Turan O. (2022). Application of genetic algorithm in exergy and sustainability: A case of aero-gas turbine engine at cruise phase. Energy.

[20] Osegi E.N., Jagun Z.O.O., Chujor C.C., Anireh V.I.E., Wokoma B.A., Ojuka O. (2023). An evolutionary programming technique for evaluating the effect of ambient conditions on the power output of open cycle gas turbine plants—A case study of Afam GT13E2 gas turbine. Appl. Energy.

[21] Tsoutsanis E., Qureshi I., Hesham M. (2023). Performance diagnostics of gas turbines operating under transient conditions based on dynamic engine model and artificial neural networks. Eng. Appl. Artif. Intell..

[22] Vladov S., Shmelov Y., Yakovliev R. Modified Searchless Method for Identification of Helicopters Turboshaft Engines at Flight Modes Using Neural Networks. Proceedings of the 2022 IEEE 3rd KhPI Week on Advanced Technology.

[23] Tayarani-Bathaie S.S., Khorasani K. (2015). Fault detection and isolation of gas turbine engines using a bank of neural networks. J. Process Control.

[24] Shen Y., Khorasani K. (2020). Hybrid multi-mode machine learning-based fault diagnosis strategies with application to aircraft gas turbine engines. Neural Netw..

[25] Vladov S., Yakovliev R., Hubachov O., Rud J. (2024). Neuro-Fuzzy System for Detection Fuel Consumption of Helicopters Turboshaft Engines. CEUR Workshop Proc..

[26] Hanachi H., Liu J., Mechefske C. (2018). Multi-mode diagnosis of a gas turbine engine using an adaptive neuro-fuzzy system. Chin. J. Aeronaut..

[27] Iliescu S.S., Făgărăşan I., Popescu V., Soare C. (2008). Gas Turbine Modeling For Load-Frequency Control. UPB Sci. Bull. Ser. C Electr. Eng. Comput. Sci..

[28] Delgado-Reyes G., Guevara-Lopez P., Loboda I., Hernandez-Gonzalez L., Ramirez-Hernandez J., Valdez-Martinez J.-S., Lopez-Chau A. (2020). State Vector Identification of Hybrid Model of a Gas Turbine by Real-Time Kalman Filter. Mathematics.

[29] Lu F., Ju H., Huang J. (2016). An improved extended Kalman filter with inequality constraints for gas turbine engine health monitoring. Aerosp. Sci. Technol..

[30] Vladov S., Shmelov Y., Yakovliev R., Petchenko M. (2023). Helicopters Turboshaft Engines Parameters Identification Using Neural Network Technologies Based on the Kalman Filter. Commun. Comput. Inf. Sci..

[31] Guo W., Qi B., Ren X., Chen H., Chen X. (2024). Vision-based closed-loop robotic fine grinding of aeroengine turbine seals utilizing Gabor Wavelet Transform. Mech. Syst. Signal Process..

[32] Han X., Huang J., Zhou X., Zou Z., Lu F., Zhou W. (2024). A novel, reduced-order optimization method for nonlinear model correction of turboshaft engines. J. Mech. Sci. Technol..

[33] Chen M., Chen H., Zhang H. (2024). Central cone film cooling scheme of turbofan engine based on multi-fidelity simulation. Appl. Therm. Eng..

[34] Vasiliev V., Zhernakov S., Muslukhov I. (2019). On-board algorithms for control of GTE parameters based on neural network technology. Bull. USATU.

[35] Pogorelov G.I., Kulikov G.G., Abdulnagimov A.I., Badamshin B.I. (2017). Application of Neural Network Technology and High-performance Computing for Identification and Real-time Hardware-in-the-loop Simulation of Gas Turbine Engines. Procedia Eng..

[36] Vladov S., Shmelov Y., Yakovliev R., Petchenko M., Drozdova S. Neural Network Method for Helicopters Turboshaft Engines Working Process Parameters Identification at Flight Modes. Proceedings of the 2022 IEEE 4th International Conference on Modern Electrical and Energy System (MEES).

[37] Wei Z., Ma Y., Yang N., Ruan S., Xiang C. (2023). Reinforcement learning based power management integrating economic rotational speed of turboshaft engine and safety constraints of battery for hybrid electric power system. Energy.

[38] Sha Y., Zhao J., Luan X., Liu X. (2024). Fault feature signal extraction method for rolling bearings in gas turbine engines based on threshold parameter decision screening. Measurement.

[39] Vladov S., Yakovliev R., Hubachov O., Rud J., Stushchanskyi Y. (2024). Neural Network Modeling of Helicopters Turboshaft Engines at Flight Modes Using an Approach Based on “Black Box” Models. CEUR Workshop Proc..

[40] Wu X., Hu X., Xiang X., Lin S., You J., Tian F. (2023). An analysis approach for micro gas turbine engine’s performance by experiment and numerical simulation. Case Stud. Therm. Eng..

[41] Yang Y., Nikolaidis T., Jafari S., Pilidis P. (2024). Gas turbine engine transient performance and heat transfer effect modelling: A comprehensive review, research challenges, and exploring the future. Appl. Therm. Eng..

[42] Gao Y., Fu J., Chen X. (2024). Correcting Hardening Artifacts of Aero-Engine Blades with an Iterative Linear Fitting Technique Framework. Sensors.

[43] Cihangir S.A., Aygun H., Turan O. (2022). Energy and performance analysis of a turbofan engine with the aid of dynamic component efficiencies. Energy.

[44] Sheng H., Chen Q., Li J., Jiang W., Wang Z., Liu Z., Zhang T., Liu Y. (2020). Research on dynamic modeling and performance analysis of helicopter turboshaft engine’s start-up process. Aerosp. Sci. Technol..

[45] Filippone A., Bojdo N. (2010). Turboshaft engine air particle separation. Prog. Aerosp. Sci..

[46] Sina Tayarani-Bathaie S., Sadough Vanini Z.N., Khorasani K. (2014). Dynamic Neural Network-Based Fault Diagnosis of Gas Turbine Engines. Neurocomputing.

[47] Vladov S., Shmelov Y., Yakovliev R. (2022). Method for Forecasting of Helicopters Aircraft Engines Technical State in Flight Modes Using Neural Networks. CEUR Workshop Proc..

[48] ElSaid A., Wild B., Higgins J., Desell T. Using LSTM Recurrent Neural Networks to Predict Excess Vibration Events in Aircraft Engines. Proceedings of the 2016 IEEE 12th International Conference on e-Science (e-Science).

[49] Tayarani-Bathaie S.S., Vanini Z.N.S., Khorasani K. Fault Detection of Gas Turbine Engines Using Dynamic Neural Networks. Proceedings of the 2012 25th IEEE Canadian Conference on Electrical and Computer Engineering (CCECE).

[50] Pan M., Wang H., Zhang C., Xu Y. (2024). Fuzzy Control for Aircraft Engine: Dynamics Clustering Modeling, Compensation and Hardware-in-Loop Experimental Verification. Aerospace.

[51] Kobayashi T., Simon D.L. (2005). Hybrid Neural-Network Genetic-Algorithm Technique for Aircraft Engine Performance Diagnostics. J. Propuls. Power.

[52] Zheng Q., Zhang H., Li Y., Hu Z. (2018). Aero-Engine On-Board Dynamic Adaptive MGD Neural Network Model Within a Large Flight Envelope. IEEE Access.

[53] Leoshchenko S.D., Pukhalska H.V., Subbotin S.A., Oliinyk A.O., Gofman Y.O. (2022). Neural network diagnostics of aircraft parts based on the results of operational processes. Radio Electron. Comput. Sci. Control.

[54] Sharma K.K., Jee G., Rajeev U.P., Padmakumar E.S. (2022). Modeling of the Helicopter Underslung Aircraft’s Lateral-Directional Dynamics. IFAC-Pap..

[55] Gao H., He A., Gao Z., Na Y., Deng Y. (2019). Flight dynamics characteristics of canard rotor/wing aircraft in helicopter flight mode. Chin. J. Aeronaut..

[56] Wei W. (2014). Identification method for helicopter flight dynamics modeling with rotor degrees of freedom. Chin. J. Aeronaut..

[57] Vladov S., Scislo L., Sokurenko V., Muzychuk O., Vysotska V., Osadchy S., Sachenko A. (2024). Neural Network Signal Integration from Thermogas-Dynamic Parameter Sensors for Helicopters Turboshaft Engines at Flight Operation Conditions. Sensors.

[58] Yan S., Shi J., Li G., Hao C., Wang Y., Yu H., Zhou W. (2024). Advances in Aeroengine Cooling Hole Measurement: A Comprehensive Review. Sensors.

[59] Vysotska V., Vladov S., Yakovliev R., Yurko A. (2024). Hybrid Neural Network Identifying Complex Dynamic Objects: Comprehensive Modelling and Training Method Modification. CEUR Workshop Proc..

[60] Jornet M. (2024). On the Cauchy-Kovalevskaya theorem for Caputo fractional differential equations. Phys. D Nonlinear Phenom..

[61] Dvirnyk Y., Pavlenko D., Przysowa R. Determination of serviceability limits of a turboshaft engine by the criterion of blade natural frequency and stall margin. Proceedings of the 9th EASN International Conference on “Innovation in Aviation & Space”.

[62] Lyantsev O.D., Kazantsev A.V., Vasin A.S. (2015). Method for identification of transfer functions of gas generator. Innov. Sci..

[63] Nako J., Psychalinos C., Elwakil A.S. (2023). One active element implementation of fractional-order Butterworth and Chebyshev filters. AEU—Int. J. Electron. Commun..

[64] Kerler M., Schäffer C., Eberhardt W., Gümmer V. Experimental Investigation of an Engine Quick-Start System with Compact Air Supply for Rotorcraft Application. Proceedings of the 17th International Symposium on Transport Phenomena and Dynamics of Rotating Machinery (ISROMAC2017).

[65] Vladov S., Shmelov Y., Yakovliev R., Petchenko M. (2023). Neural Network Method for Parametric Adaptation Helicopters Turboshaft Engines On-Board Automatic Control System Parameters. CEUR Workshop Proc..

[66] Takenaga S., Ozaki Y., Onishi M. (2023). Practical initialization of the Nelder–Mead method for computationally expensive optimization problems. Optim. Lett..

[67] Gao F., Han L. (2012). Implementing the Nelder-Mead simplex algorithm with adaptive parameters. Comput. Optim. Appl..

[68] Zhao J., Zhang J., Li Y., Wu Y. (2021). A Control Design of Rotor Speed Regulation for an Aero-engine based on Smooth Switching Strategy. IFAC-Pap..

[69] Feng H., Liu B., Xu M., Li M., Song Z. (2024). Model-based deduction learning control: A novel method for optimizing gas turbine engine afterburner transient. Energy.

[70] Liu Y., Jafari S., Nikolaidis T. (2021). Advanced optimization of gas turbine aero-engine transient performance using linkage-learning genetic algorithm: Part I, building blocks detection and optimization in runway. Chin. J. Aeronaut..

[71] Catana R.M., Dediu G. (2023). Analytical Calculation Model of the TV3-117 Turboshaft Working Regimes Based on Experimental Data. Appl. Sci..

[72] Gebrehiwet L., Nigussei Y., Teklehaymanot T. (2022). A Review-Differentiating TV2 and TV3 Series Turbo Shaft Engines. Int. J. Res. Publ. Rev..

[73] Vladov S.I., Moskalyk V.M., Siora A.S., Dieriabina I.O., Gvozdik S.D. (2020). Analysis of TV3-117 aircraft engine dynamics using Elman’s neural network. Visnyk Kherson Natl. Tech. Univ..

[74] Liu Z., Ning D., Hou J. (2024). A novel Elman neural network based on Gaussian kernel and improved SOA and its applications. Expert Syst. Appl..

[75] Huang J., Qin R. (2024). Elman neural network considering dynamic time delay estimation for short-term forecasting of offshore wind power. Appl. Energy.

[76] Lila V.B., Puchkov E.V. (2014). Methodology of training recurrent artificial neural network with dynamic stack memory. Softw. Syst..

[77] Vladov S., Yakovliev R., Vysotska V., Nazarkevych M., Lytvyn V. (2024). The Method of Restoring Lost Information from Sensors Based on Auto-Associative Neural Networks. Appl. Syst. Innov..

[78] Vladov S., Yakovliev R., Bulakh M., Vysotska V. (2024). Neural Network Approximation of Helicopter Turboshaft Engine Parameters for Improved Efficiency. Energies.

[79] Vladov S., Shmelov Y., Yakovliev R., Stushchankyi Y., Havryliuk Y. (2023). Neural Network Method for Controlling the Helicopters Turboshaft Engines Free Turbine Speed at Flight Modes. CEUR Workshop Proc..

[80] Balakrishnan N., Voinov V., Nikulin M.S., Balakrishnan N., Voinov V., Nikulin M.S. (2013). Chapter 2—Pearson’s Sum and Pearson-Fisher Test. Chi-Squared Goodness of Fit Tests with Applications.

[81] Avram F., Leonenko N.N., Šuvak N. (2012). Hypothesis testing for Fisher–Snedecor diffusion. J. Stat. Plan. Inference.

[82] Babichev S., Krejci J., Bicanek J., Lytvynenko V. Gene expression sequences clustering based on the internal and external clustering quality criteria. Proceedings of the 2017 12th International Scientific and Technical Conference on Computer Sciences and Information Technologies (CSIT).

[83] Hu Z., Kashyap E., Tyshchenko O.K. (2022). GEOCLUS: A Fuzzy-Based Learning Algorithm for Clustering Expression Datasets. Lect. Notes Data Eng. Commun. Technol..

[84] Golovko V., Savitsky Y., Laopoulos T., Sachenko A., Grandinetti L. Technique of learning rate estimation for efficient training of MLP. Proceedings of the IEEE-INNS-ENNS International Joint Conference on Neural Networks. IJCNN 2000. Neural Computing: New Challenges and Perspectives for the New Millennium.

[85] Liao Z., Zhan K., Zhao H., Deng Y., Geng J., Chen X., Song Z. (2024). Addressing class-imbalanced learning in real-time aero-engine gas-path fault diagnosis via feature filtering and mapping. Reliab. Eng. Syst. Saf..

[86] Gus’kov S.Y., Lyovin V.V. (2015). Confidence interval estimation for quality factors of binary classifiers—ROC curves, AUC for small samples. Eng. J. Sci. Innov..

[87] Anfilets S., Bezobrazov S., Golovko V., Sachenko A., Komar M., Dolny R., Kasyanik V., Bykovyy P., Mikhno E., Osolinskyi O. (2020). Deep multilayer neural network for predicting the winner of football matches. Int. J. Comput..

[88] Cherrat E.M., Alaoui R., Bouzahir H. (2020). Score fusion of finger vein and face for human recognition based on convolutional neural network model. Int. J. Comput..

[89] Morozov V.V., Kalnichenko O.V., Mezentseva O.O. (2020). The method of interaction modeling on basis of deep learning the neural networks in complex IT-projects. Int. J. Comput..

[90] Paliy I., Sachenko A., Koval V., Kurylyak Y. Approach to Face Recognition Using Neural Networks. Proceedings of the 2005 IEEE Intelligent Data Acquisition and Advanced Computing Systems: Technology and Applications.

[91] Andriushchenko K., Rudyk V., Riabchenko O., Kachynska M., Marynenko N., Shergina L., Kovtun V., Tepliuk M., Zhemba A., Kuchai O. (2019). Processes of managing information infrastructure of a digital enterprise in the framework of the «Industry 4.0» concept. East.-Eur. J. Enterp. Technol..

[92] Romanova T.E., Stetsyuk P.I., Chugay A.M., Shekhovtsov S.B. (2019). Parallel Computing Technologies for Solving Optimization Problems of Geometric Design. Cybern. Syst. Anal..

[93] Shmelov Y., Vladov S., Klimova Y., Kirukhina M. Expert system for identification of the technical state of the aircraft engine TV3-117 in flight modes. Proceedings of the System Analysis & Intelligent Computing: IEEE First International Conference on System Analysis & Intelligent Computing (SAIC).

